# SMRT and Illumina RNA-Seq Identifies Potential Candidate Genes Related to the Double Flower Phenotype and Unveils SsAP2 as a Key Regulator of the Double-Flower Trait in *Sagittaria sagittifolia*

**DOI:** 10.3390/ijms23042240

**Published:** 2022-02-17

**Authors:** Meiping Gao, Wen Jiang, Zhicheng Lin, Qian Lin, Qinghua Ye, Wei Wang, Qian Xie, Xinhua He, Cong Luo, Qingxi Chen

**Affiliations:** 1 College of Horticulture, Fujian Agriculture and Forestry University, Fuzhou 350002, China; gmp2009@163.com (M.G.); qinghua@gxaas.net (Q.Y.); wangwei@gxaas.net (W.W.); xieqian@gxaas.net (Q.X.); 2 Biotechnology Research Institute, Guangxi Academy of Agricultural Sciences, Nanning 530007, China; jiangwen@gxaas.net (W.J.); linzhicheng@gxaas.net (Z.L.); linqian@gxaas.net (Q.L.); 3 College of Agriculture, Guangxi University, 100 Daxue Road, Nanning 530004, China; honest66222@163.com (X.H.); congluo@gxaas.net (C.L.)

**Keywords:** *Sagittaria sagittifolia*, double flower, SMRT and RNA-Seq, WGCNA, plant hormone, *APETALA2*

## Abstract

Double flowers are one of the important objectives of ornamental plant breeding. *Sagittaria sagittifolia* is an aquatic herb in the Alismataceae family that is widely used as an ornamental plant in gardens. However, the reference genome has not been published, and the molecular regulatory mechanism of flower formation remains unclear. In this study, single molecule real-time (SMRT) sequencing technology combined with Illumina RNA-Seq was used to perform a more comprehensive analysis of *S. sagittifolia* for the first time. We obtained high-quality full-length transcripts, including 53,422 complete open reading frames, and identified 5980 transcription factors that belonged to 67 families, with many MADS-box genes involved in flower formation being obtained. The transcription factors regulated by plant hormone signals played an important role in the development of double flowers. We also identified an *AP2* orthologous gene, *SsAP2*, with a deletion of the binding site for miR172, that overexpressed *SsAP2* in *S. sagittifolia* and exhibited a delayed flowering time and an increased number of petals. This study is the first report of a full-length transcriptome of *S. sagittifolia*. These reference transcripts will be valuable resources for the analysis of gene structures and sequences, which provide a theoretical basis for the molecular regulatory mechanism governing the formation of double flowers.

## 1. Introduction

Double flowers, which are characterized by the excessive development of petals, are important in ornamental flowers because they are considered to be more attractive in gardens than their normal counterparts. Breeders have selected the double flower phenotype in many varieties, such as gerbera daisy (*Gerbera jamesonii*), carnations (*Dianthus caryophyllus*), and peach (*Prunus persica*) [1,2,3]. In the past few decades, flower development has been widely studied in arabidopsis (*Arabidopsis thaliana*) and snapdragons (*Antirrhinum majus*), and the ABC model has been established to explain the characteristics of floral organs [4,5,6]. According to this model, class C genes *AG* (*AGAMOUS*) determine the formation of stamens and the fate of carpels. Many studies have shown that the loss of C function or a change in its expression is the main reason for the excessive number of petals [7,8,9,10]. For example, the loss of function of an *AG* orthologous gene (*ThtAG1*) in rue anemone (*Thalictrum thalictroides*) leads to the development of double flowers [11,12]. In the oshima cherry (*Prunus lannesiana*), the restricted expression of homologous *AG* has been proven to aid the development of double flowers [8]. Alternatively, in many horticultural plants, such as Japanese gentian (*Gentiana scabra*) [10], common camellia (*Camellia japonica*) [13], garden petunia (*Petunia hybrida*) [14], and persian cyclamen (*Cyclamen persicum*) [9], the inhibition of C genes results in the formation of double flowers.

It has also been reported that *AP2* (*APETALA2*) affects the formation of double flowers. It is a Class A gene and solely determines the formation of sepals, whereas Class A and B genes *PI* and *AP3* (*PISTILLATA* and *APETALA3*) jointly determine the formation of petals. *AP2* is a member of the euAP2 lineage, which has two AP2 DNA binding domains and one target site for the miR172 [15,16]. Except for *AP2*, all proteins encoded by the ABCDE model are MADS-box transcription factors [17,18]. The proteins encoded by these genes have a conserved MADS domain and a middle (I), keratin (K), and C-terminus. Sequence differences in the MADS-box proteins of different flowering plant species have been used to clarify the evolution and diversity of floral organ characteristics [19,20]. During the development of *A. thaliana* flowers, the inhibition of *euAP2* gene by miR172 is critical to maintain the certainty of flower organs [21]. In peach (*Prunus persica*) and rose, a mutant allele of the *AP2* transcription factor was found to be primarily related to double flowers, and this transcription factor plays a conserved role in the regulation of flower patterns and development transformation [3,17]. The *ap2* mutant has an abnormal flower phenotype in *A. thaliana*, including loss of petals and homologous transformation of sepals to carpels. Flower organs will exhibit a similar phenotype of double flowers to the *ap2* mutant after the overexpression of miR172 with the 35S promoter [22,23]. Unlike the situation in Arabidopsis, the *AP2-like* gene (*LIP1* and *LIP2*) in *Antirrhinum majus* only redundantly inhibits the function of flower class B and C genes [24]. Therefore, there are differences in the function of the *AP2* gene among different species.

In addition to the interactions between different genes, hormonal factors can also lead to an increase in the number of petals. For example, flowers of rose balsam (*Impatiens balsamina*) that have been treated with gibberellin (GA3) can change from semi-double to full double flowers [25], while the *Dianthus* ‘carnation’ variety ‘White Sim’ treated with auxin (indole-3-acetic acid, IAA) and GA3 exhibits the phenomenon of increased petals. However, it is not clear how hormones regulate related genes that contribute to the formation of flower organs.

The arrowhead plant (*Sagittaria sagittifolia* L.) with double flowers is an aquatic plant in the Alismataceae family, with a beautiful flower type and a long flowering time that lasts for half a year. It is becoming increasingly popular in gardens, and thus, it has great substantial potential for market popularization and application. However, little is currently known about the molecular regulatory mechanism involved in flower formation [16]. Single molecule real-time (SMRT) sequencing technology has been increasingly applied to transcriptome analysis in many species [26,27]. To study the molecular mechanism of the development and formation of double flowers in more detail, we analyzed the expression of genes in different tissues between single and double flower varieties of *S. sagittifolia*. To our knowledge, this study is the first report of the full-length transcriptome of the aquatic ornamental plant *S. sagittaria*. We also identified an *AP2* orthologue gene (*SsAP2*), and the *SsAP2* transgenic *S. sagittifolia* exhibited stamens and carpels that were petalized and formed a semi-double flower phenotype, indicating that *SsAP2* plays an important role in the formation of double flowers. These reference transcripts will be valuable resources for the analysis of various gene structures and sequences, which provide a theoretical basis for the molecular regulatory mechanism of the development of double flowers.

## 2. Results

### 2.1. Floral Morphological Characteristics of Single and Double Flowers of S. sagittifolia

**Figure 1:**
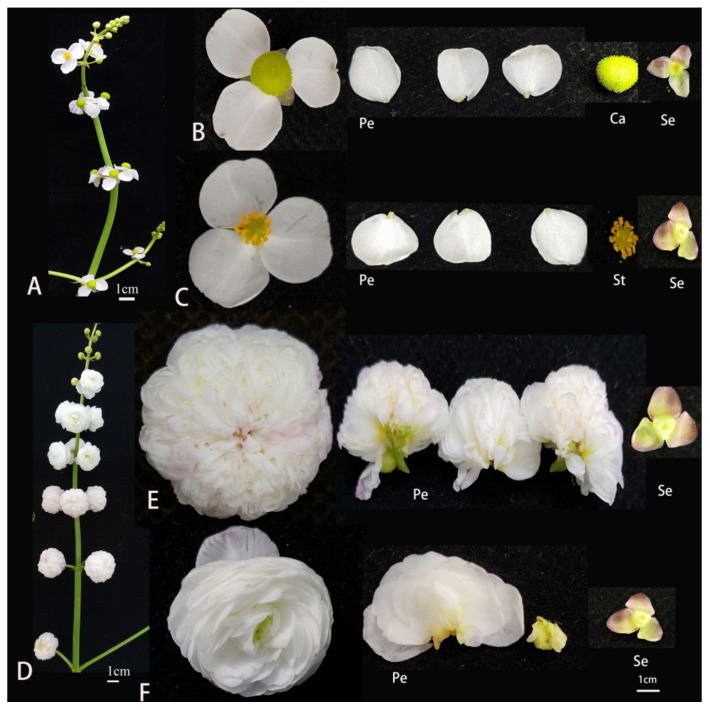
Phenotypic map of single and double flowers of *S. sagittifolia*. Scale bar = 1 cm. (**A**) Inflorescences of single *S. sagittifolia* (SF). (**B**) A female flower of single *S. sagittifolia*, and (**C**) a male flower of single *S. sagittifolia*. (**D**) Inflorescences of double *S. sagittifolia* (DF). (**E**) A female flower of double *S. sagittifolia*, and (**F**) a male flower of double *S. sagittifolia*. Abbreviations: Se, sepals; Pe, petals; Ca, carpels; St, stamens.

Wild-type *S. sagittifolia* is a racemose inflorescence, unisexual, and monoecious (Figure 1A). Female flowers appear earlier and are primarily concentrated in the first to third whorls of inflorescence at the base. The male flowers appear later with many whorls at the top of the inflorescence. There are two bracteoles and three sepals outside of the female and male flowers. In single flowers, there are three petals colored yellow and white and numerous carpels in female flowers (Figure 1B), and 15–18 stamens in male flowers (Figure 1C). The characteristics of floral morphology in single and double flowers are similar, except for the petal morphology (Figure 1D). In double female flowers, 200–280 petals are produced instead of carpels (Figure 1E); in double male flowers, 60–90 petals are produced instead of stamens, and the petals of male flowers are slightly larger than those of the female flowers (Figure 1F). To describe the characteristics in the development of *S. sagittifolia*, we compared the flower organs of single and double flowers. Single flowers can be both male and female with sepals, petals, and stamens or carpels, which were observed from the first to third whorl (Figure 1B,C). Similar to the single flower, the first and second whorls of double flowers possessed normal sepals and petals, but the third whorl stamens of the double male flowers were missing, while the carpels of female flowers were missing (Figure 1E,F). The flower timing of double flowers is later than that in single flowers.

### 2.2. Observation the Development of Single and Double Flowers Morphology of S. sagittifolia under Light Microscope

**Figure 2:**
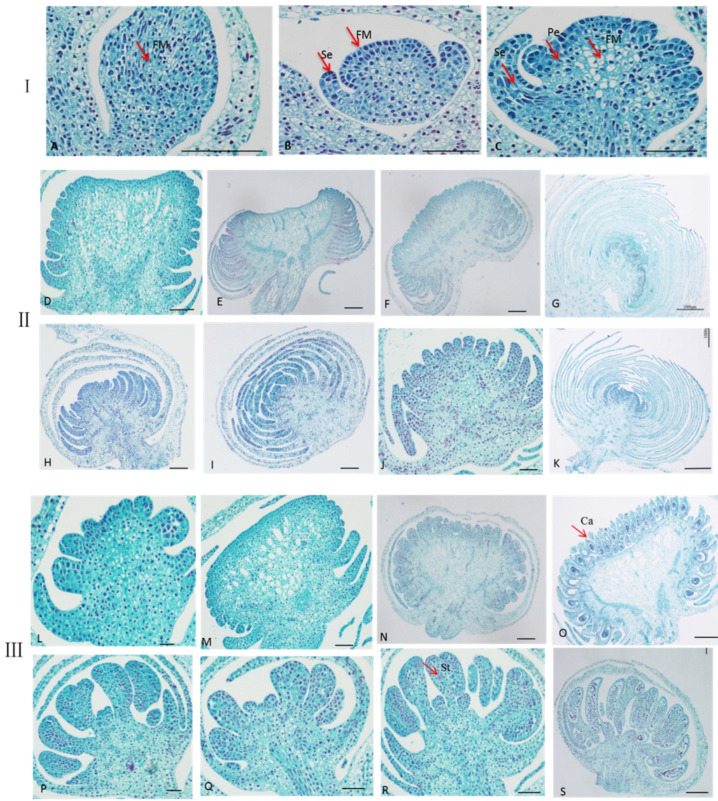
Observation of the development of single and double flowers’ morphology of S. sagittifolia under optical microscope. Scale bar = 1000 μm. Section I (**A**–**C**): The development of sepal and petal formation in single and double flowers. (A) The floral meristem was formed. (**B**) The sepal primordial was produced, and (**C**) the petal primordial was produced. Section II (**D**–**K**): Flower growth in double flowers. (**D**–**G**) The continuous growth of the petal in double female flowers. (**H**–**K**) The continuous growth of the petal in double male flowers. Section III (**L**–**S**): Flower growth in single flowers. (**L**–**Q**) Petals continuously grow and carpels are produced, (**P**–**S**) petals continuously grow, and stamens are produced.

Conventional paraffin sectioning with the aid of light microscopy in single and double flowers of *S. sagittifolia* at different stages of development are shown in Figure 2. The paraffin sections indicated that when the flower buds of *S. sagittifolia* began to differentiate, the floral meristem cells became active and enlarged. The top became wider and hemispherical (Figure 2A). The floral meristem developed further, and the transverse division was larger than that in other directions, the appearance of the subsequent sepal primordium (Figure 2B). With further growth, then petal primordial produced (Figure 2C). In these stages, the organ primordium development of double flowers is basically the same as that of single flowers. The petal primordium continuously grew in double female flowers (Figure 2D–G) and male flowers (Figure 2H–K). Different from double flowers, in a single female flower, the carpel primordia began to occur at the epidermal cells inside the petal base, and the organs of flower buds began to develop and continuously grow (Figure 2L–O). In the male flower, the carpel primordia began to occur at the epidermal cells inside the petal base, and the organs of flower buds continuously grew (Figure 2P–S).

Based on the observation of single and double flowers of *S. sagittifolia* under optical microscope and floral physiology, the flower buds of *S. sagittifolia* were divided into three key stages of flower bud differentiation: (1) sepal differentiation (Figure 2B); (2) petal differentiation (Figure 2C); and (3) stamen (pistil) differentiation (Figure 2E). Paraffin sections showed that there were normal carpels and stamens in the single flowers (Figure 2L–S), while the petals were produced instead of carpels and stamens in double flowers (Figure 2D–K) during the early stage of flower bud differentiation.

### 2.3. Sequencing Data Statistical Analysis of SMRT and Illumina RNA-Seq

A total of 47.73 Gb of clean data was obtained using Pacific Biosciences Iso-Seq. The transcriptome sequencing of 34 cDNA libraries was completed, and a total of 238.53 Gb of clean data was obtained with Q30 > 92% (Supplementary Table S2-1). Categorization information for transcripts in *S. sagittifolia* from the SMRT data is shown in Supplementary Tables S2-2 and S2-3. There are 656,275 circular consensus (CCS) readings with a mean read length of 2234 bp that were obtained, and the length distribution is shown in Supplementary Figure S2A. A total of 579,295 full-length non-chimeric (FLNC) sequences were obtained, accounting for 88.27% of the CCS readings, and the read length distribution for each size bin is shown in Supplementary Figure S2B. The full-length non-chimeric sequences were clustered to obtain 156,338 consistent sequences, with an average length of 2208 bp. A total of 153,620 consistent sequences of high quality were obtained by polishing the consistent sequences, which comprised 98.26%. The RNA-Seq transcriptome data were used to correct the low-quality consistent sequences, and 65,633 transcripts were obtained by combining and eliminating redundancy. The degree of completeness assessment was nearly 88% (Supplementary Figure S2C). The transcriptome in this study exhibited a long transcript sequence length and high assembly integrity. A total of 53,422 CDS were identified.

The TransDecoder program was used to predict 63,162 open reading frames (ORFs). The length distribution of the predicted complete ORF coding protein sequence is shown in Supplementary Figure S2D. There are 12,425 transcripts distributed in the range of 100–200 bp. To study the differences in transcription between the double and single flower development in *S. sagittifolia*, we conducted a systematic clustering analysis on the average fragments per kilobase of transcript per million mapped fragments (FPKM) values of all the genes that were expressed in 34 tissue samples. Highly correlated groups in these analyses were clustered together (Supplementary Figure S2E). As expected, there were two groups for the flower bud transcriptome of the two varieties, with the exception of the first stage of single female flowers. There was one group for the two varieties at the third stage (S3) of early flower development, and another at S1 and S2. Different varieties were clustered into two groups, and there were two groups for female flowers and male flowers of each variety. The results showed that there was satisfactory repeatability for the samples, and there were substantial differences in transcription between the two varieties during their early flower development.

**Figure 3:**
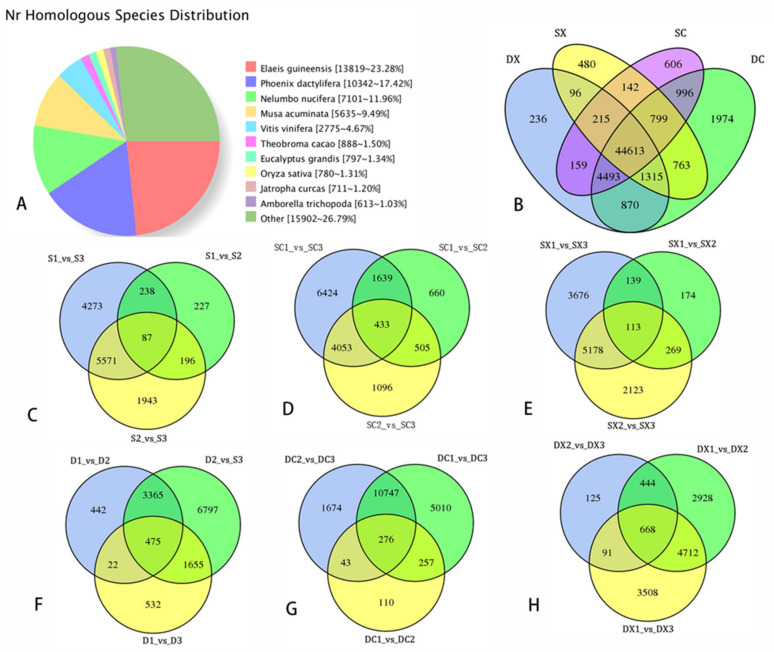
Nr homologous species distribution and a Venn diagram of differentially expressed genes at different developmental stages. Abbreviations: DX, male double flower; DC, female double flower; SX, male single flower; and XC, male single flower; differentially expressed in a Venn diagram. (**A**) Nr homologous species distribution; (**B**) Venn diagram of female and male in single and double flowers; (**C**) Venn diagram of three developmental stages for single flowers; (**D**) Venn diagram of three developmental stages for single female flowers; (**E**) Venn diagram of three developmental stages for single male flowers, (**F**) Venn diagram of the three developmental stages for double male flowers; (**G**) Venn diagram of the three developmental stages for double female flowers; (**H**) Venn diagram of the three developmental stages for double male flowers.

A total of 2062 lncRNAs were identified. Four different methods (CPC2, Cpat, PLEK, and CNCI) were used to predict the domain analysis and evaluate the coding potential. The 2062 predicted lncRNA sequences were considered to be target genes. A total of 675 lncRNAs predicted the target genes (Supplementary Table S3). In our research, a total of 4636 alternative splicing (AS) events were predicted. As there is no reference genome for *S. sagittifolia*, the type of AS could not be identified. According to the KEGG enrichment, these genes are highly enriched in gluconeogenesis (147), splicer (129), carbon metabolism (129), protein processing in endoplasmic reticulum (109), and amino acid biosynthesis. Transcripts > 500 bp were analyzed to determine the density distribution of the different SSR types. Compound SSR was 16.09 per Mb, and the sequence densities of those that contained > 1 SSR were 32, 34.60, 46, and 57.84 per Mb. The statistics regarding data filtering from the RNA-Seq data for *S. sagittifolia* are shown in Supplementary Table S4. The results showed that an average of 86.45% clean sequences were compared to full-length transcripts. A comparison of the Nr results for distribution of homologous species is shown in Figure 3A. The largest number of annotations were for African palm (*Elaeis guineensis*, 23.28%), followed by date palm (*Phoenix dactylifera*, 17.42%), and sacred lotus (*Nelumbo nucifera*, 11.96%). *Nelumbo nucifera* is an aquatic floral plant that may closely resemble *S. sagittifolia.* Cavendish banana (*Musa acuminata*) also has many annotated homologous sequences, with a total of 5635 homologous sequences, accounting for 9.49%. There were a few homologous sequences, e.g., grapes (*Vitis vinifera*, 4.67%), cacao (*Theobroma cacao*, 1.5%), rose gum (*Eucalyptus grandis*, 1.34%), rice (*Oryza sativa*, 1.31%), and physic nut (*Jatropha curcas*, 1.03%).

### 2.4. Differential Expression and Function Annotation of Transcripts Obtained by Transcriptome Sequencing

We compared the transcripts of female and male flowers in single and double *S. sagittifolia* during three different development stages (a total of 34 samples). The level of gene expression was calculated and normalized to the FPKM value. A total of 25,079 differential genes were identified. There were 44,613 genes in common; 236, 480, 606, and 1974 DEGs were unique in DX (double male), DC (double female), SX (single male), and XC (single male), respectively (Figure 3B). In the comparison group of SC1-VS-SC3, SX1-VS-SX3, DC1-VS-DC3, and DX1-VS-DX3, 6242, 3676, 5010, and 3508 DEGs were detected, respectively (Figure 3C–H)). The results showed that the most DEGs were in S1-vs-S3, and there were more DEGs in female flowers than those in male flowers. The database function is noted, and the number of transcripts annotated in the set are shown in Supplementary Table S5.

A total of 24,381 transcripts (40.78%) were assigned to COG for functional classification, which were divided into 25 functional categories (Supplementary Figure S3). The top five categories are as follows: ‘General Function Prediction only’ had 2864, with the largest number, accounting for 18.99%; ‘Translation, ribosomal structure and biogenesis’ accounted for 10.28%; ‘Signal transduction mechanisms’ accounted for 9.43%; ‘Carbohydrate transport and metabolism’ accounted for 8.94%; ‘Protein turnover (Chaperones)’ accounted for 7.8%; and ‘Posttranslational modification’ accounted for 7.04%. Thus, in addition to the general functions and the most basic life activities, signal transduction and posttranslational modification play an important role in the development of *S. sagittifolia*.

**Figure 4:**
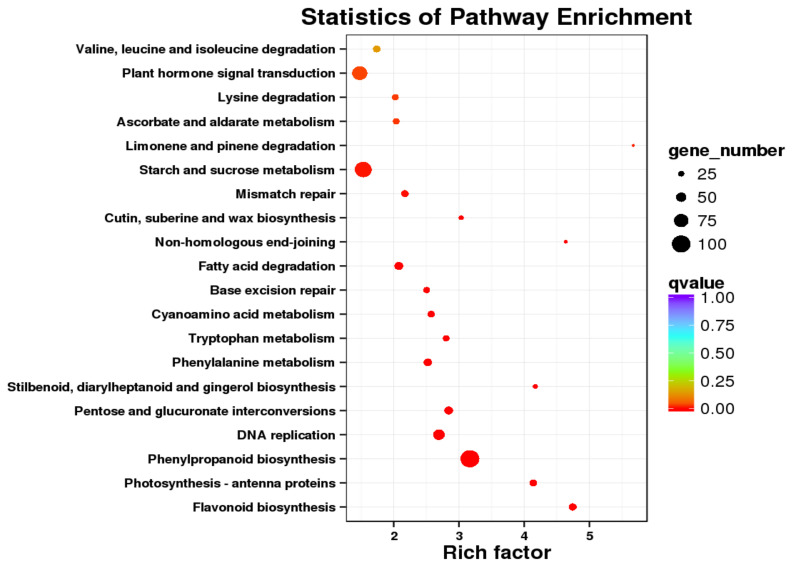
The top 20 enrichment pathways of DEGs.

A total of 42,566 transcripts (71.17%) were annotated to the Gene Ontology (GO) database, which were distributed in three main categories: ‘biological processes’, ‘cellular components’ and ‘molecular functions’, and divided into 47 subcategories (Supplementary Figure S4). In the biological processes, the focus was on ‘cellular processes’, ‘metabolic process’, ‘single-organism process’ and ‘response to stimulus’. During the process of cell activity, the focus was on ‘the formation of cell’, ‘cell part’, and ‘organelle and membrane’. In the molecular function, most genes were significantly enriched in ‘catalytic activity’, ‘binding’, ‘transporter activity’ and ‘structural molecule activity’. DEGs participate in more than 110 types of metabolism during the flower development of *S. sagittifolia*. The top 20 enrichment pathways are shown in Figure 4, and the DEGs were primarily enriched in ‘ribosome’, ‘carbon metabolism’, ‘spliceosome’, ‘starch and sucrose metabolism’, ‘plant hormone signal transduction’, and ‘phenylpropanoid biosynthesis’. These DEGs may play an important role in the development of *S. sagittifolia*.

During the development of the three stages, there were 103 MADS genes and 24 *AP2*-like differentially expressed that were related to flower development in the two varieties (Supplementary Table S6). Compared with single flowers, most of these genes were downregulated in double flowers (Table S6). Many DEGS related to plant hormones were also identified, including auxin (IAA), cytokinin (CK), gibberellic acid (GA), and jasmonic acid (JA). These DEGs may play an important role in the development of *S. sagittifolia* flowers.

### 2.5. Transcription Factors and WGCNA

**Figure 5:**
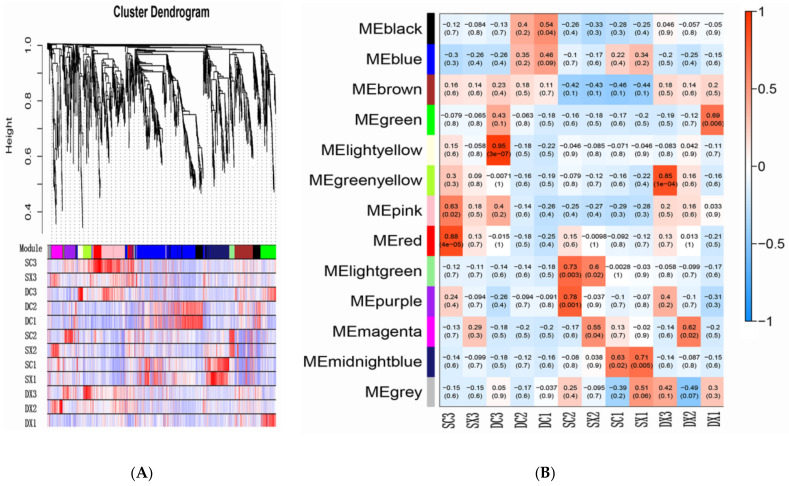
Transcription factor (TF) with a weighted gene co-expression network analysis (WGCNA): (**A**) shows cluster dendrogram for TFs; (**B**) shows module trait related to TFs. Each leaf on the tree represents a gene. The different modules of the trunk branch are marked with different colors. The module name is displayed on the left, with each row corresponding to a module and each column corresponding to a sample. Each cell uses a different color to represent the correlation coefficient between the module and the sample, and the high correlation is expressed in red.

Transcription factors (TFs) play an important role in plant growth and development. We found 5980 transcription factors from 67 families in the transcriptome (Supplementary Table S7). Most transcripts are members of bHLH (216), MYB (241), NAC (270), B3 (186), C2H2 (162), bZIP (136), C3H (138), AP2/ERF-ERF (122), and MADS (103). These factors are widely involved in plant growth and are related to flower development and hormone responses. We used weighted gene co-expression network analysis (WGCNA) to further explore the relationship between the TFs (TFs with a FPKM value < 1 and K-ME < 0.7 filtered) and traits. Highly correlated TF clusters are defined as modules, and the WGCNA identified 12 different modules, and the different modules of the trunk branch are marked with different colors (Figure 5A). The colors blue (485 TF), black (155 TF), brown (145 TF), green (115 TF), midnight blue (172 TF), pink (205 TF), and red (94 TF) are rich in these modules.

Most modules were related to > 1 tissue sample or developmental stage, while a few modules were only related to specific tissues or flower development stages. Three expression modules were highly correlated with the development of *S. sagittifolia* flowers (r ≥ 0.85) (Figure 5B). These modules contain many genes that are related to protein processing endoplasmic reticulum (ko 04141), plant hormone signal transduction (ko 04075), and starch and sucrose metabolism (ko 00500). There was a particular correlation between Red and SC3 (0.88), and this module contains the highest number of AP2, WRKY, bHLH, and MADS-box transcription factors. Notably, each module is primarily enriched in four KEGG pathways, including plant hormone signal transduction (ko 04075), spliceosome (hsa03040), genetic information processing, and plant–pathogen interaction (ko 04626). Based on the module gene enrichment analysis, it is worth noting that the blue module is rich in the highest number of plant hormone signal transductions, which highly correlates with the development of female and male flowers in double flowers. Based on the review, the results of transcription factor analysis show that in addition to flower development-related transcription factors, plant hormone signal-regulated transcription factors played an important role in the development of double flowers.

### 2.6. Identification of DGEs Related to Flower Formation and Verification of Gene Expression by qRT-PCR

A WGCNA of the DGEs was conducted to obtain the WGCNA score. In this study, based on the pattern of gene expression, 22 modules were identified by WGCNA, among which several modules showed functional specificity at different stages (marked with different colors). This shows a correlation coefficient between the characteristic genes of each module in 22 different modules, and each different sample (trait) can be observed in Supplementary Figure S6. Notably, based on the module gene enrichment analysis, IAA and GA3 traits were significantly correlated with the MEyellowgreen module and MEgreen module (R2 > 0.8, *p* < 1.04). There are four co-expression modules (magenta, coral1, darkslateblue, and plum1) that were highly correlated with the development of *S. sagittifolia* flowers (r ≥ 0.91) (Figure S7). Hub genes were identified based on the criteria of characteristic gene connectivity (KME) ≥ 0.98 and edge weight ≥ 0.5. In each network module, the hub genes are highly connective with the other genes. They occupy a central position in the network cluster, and thus, they are considered to be an important part of the network. A total of 68 DEGs were identified as hub genes, and these hub genes were significantly enriched and analyzed by the KEGG pathway using a hypergeometric test. The results showed that starch and sucrose metabolism, photosynthesis, and signal transduction were the dominant genes related to flower development.

To verify the accuracy of the RNA-Seq results, we conducted qRT-PCR verification experiments on three biological repetitive sequences. We initially chose the high expression of DEGs and with differences in expression at different developmental stages. Based on most of the similar expression trends of genes and homologous genes, we then selected the one with the highest expression level and the most significant difference for fluorescence quantitative analysis. Six representative DEGs (*AP2*, *AG*, *AP3*, *SEP*, *PI*, and *WUS*) related to flower development were selected for qRT-PCR analysis (the primers are shown in Supplementary Table S2). The patterns of expression as revealed by RNA-Seq for all six DEGs were consistent with the qRT-PCR data, which indicated that the sequencing data were reliable.

**Figure 6:**
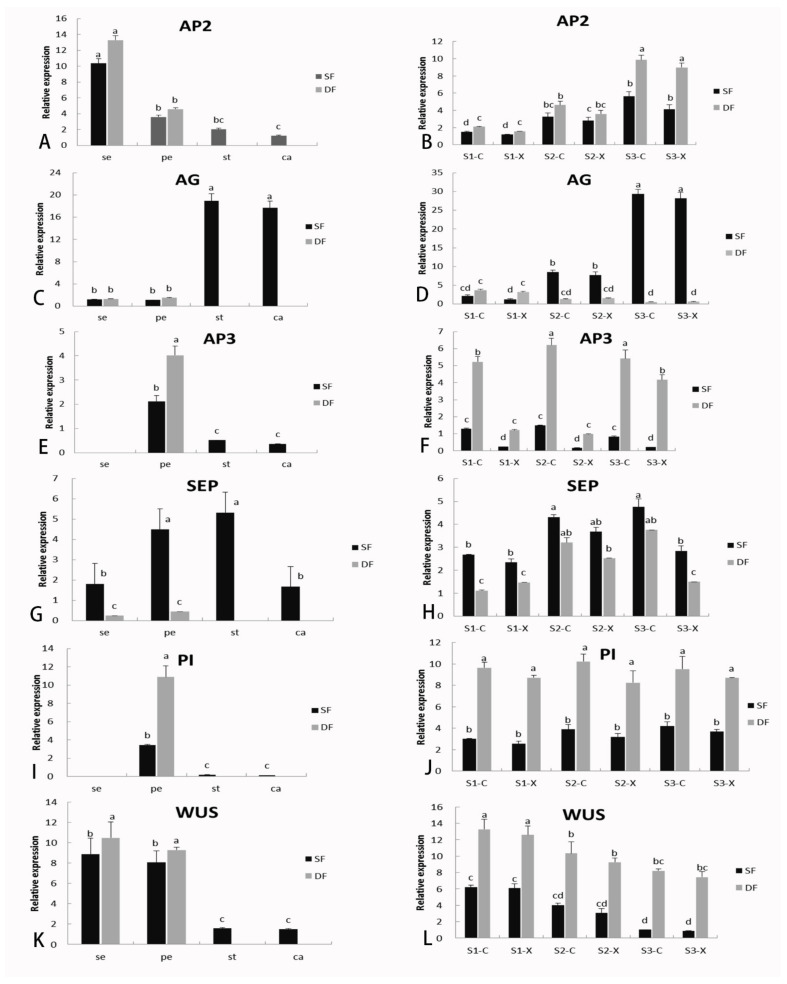
Differential genes in the floral organs and floral development stages shown by RT-PCR. The left half of the figure (**A**,**C**,**E**,**G**,**I**,**K**) shows the expression of differential genes of AP2, AG, AP3, SEP, PI, WUS respectively in flower organs: sepals (se), petals (pe), stamens (st), and carpels (ca), while the right half (**B**,**D**,**F**,**H**,**J**,**L**) shows the expression of differential genes in the three stages of the early development of single and double flowers. Lowercase letters indicate significant differences. The following is the same.

As shown in Figure 6, *AP2* and *AG* were significantly differentially expressed during the three stages of early development of flower buds. The specific expression results are as follows: the expression of *AP2* in the first whorl of the sepal was significantly higher than that in the carpel and stamen (Figure 6A). With the development of flowers, the expression of *AP2* increased, and the expression in double flowers was higher than that in single flowers, with no significant differences between female and male flowers (Figure 6B). *AG* was primarily expressed in single flowers in stamens and carpels of the third whorls, and with the highest expression in S3. The expression was low in double flowers (Figure 6C,D).

The changes in expression of *AP3*, *PI*, and *SEP* tended to be stable. *AP3* and *PI* affiliated with class B genes were significantly expressed in the petals and slightly expressed in the stamens and carpels but not in the sepals (Figure 6E,I), and the expression in double flowers was significantly higher than that in single flowers (Figure 6F,J). *SEP* was expressed in all the flower organs of *S. sagittifolia*, although expression was particularly high in the petals and stamens (Figure 6G,H). *WUS* (W*USCHEL*) was primarily expressed in the sepals and petals (Figure 6K). In single flowers, *WUS* showed a high level of expression in S1 and a low level of expression in S3, while in double flowers, the expression of *WUS* remained at a high level during the three development stages and was significantly higher than that in single flowers (Figure 6L).

### 2.7. Hormone Biosynthesis and Signaling-Related Genes in Flower Development

**Figure 7:**
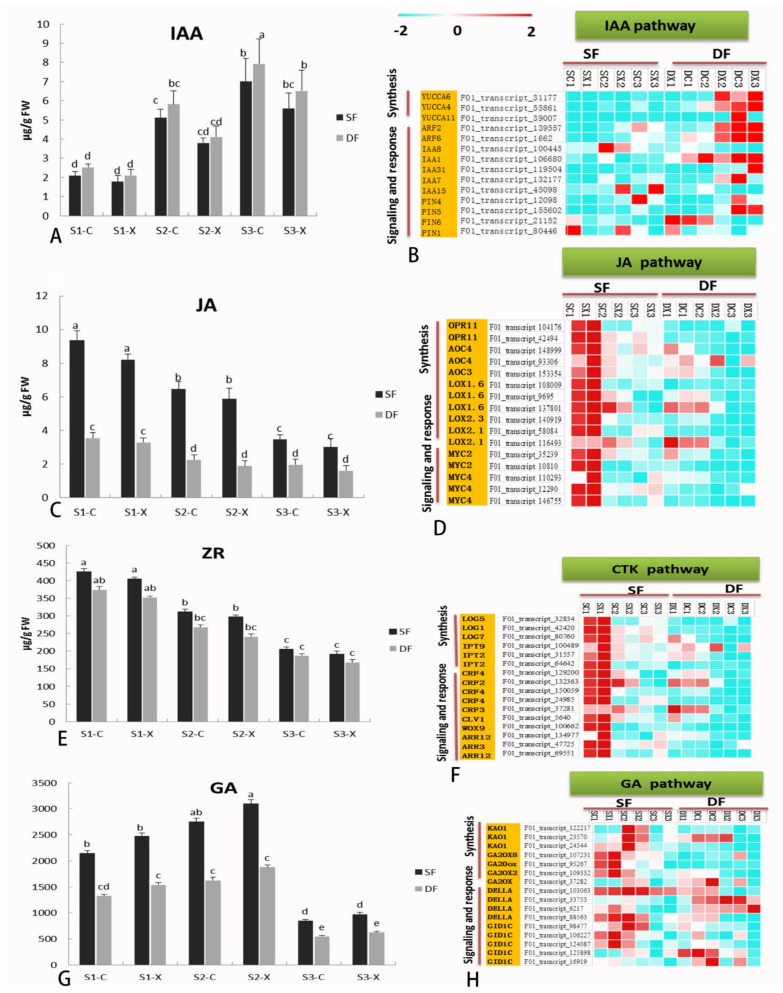
The dynamic changes in endogenous hormone content in female/male flowers and a heat map of gene expression related to hormone biosynthesis and signaling pathways in single and double flower varieties during flower development. (**A**,**C**,**E**,**G**) The changes in the endogenous hormone content of IAA, JA, ZR, and GA in single and double flowers. (**B**,**D**,**F**,**H**) Heat maps of gene expression related to IAA, JA, ZR, and GA hormone biosynthesis and signal pathways. Abbreviations: GA, gibberellin; IAA, indole acetic acid; JA, jasmonic acid; ZR, zeatin nucleoside. Lowercase letters indicate significant differences. The following is the same.

The changes of hormone levels during flower bud development of *S. sagittifolia* were also detected. We monitored the levels of endogenous auxin (AUX), zeatin nucleoside (ZR), GA, and JA in male and female *S. sagittifolia* flower buds during flower bud differentiation (Figure 7). The results of an ultra-high performance liquid chromatography–tandem mass spectrometry (UPLC–MS/MS) analysis in the three stages of the development of early flower buds showed that the lowest content measured was for IAA (Figure 7A), followed by JA (Figure 7C), and the highest amount of hormone measured was for GA (Figure 7G). GA and ZR exhibited a different trend, with ZR decreasing, while GA was ‘rising–decreasing’ (Figure 7E,G). The amount of IAA in the double flower buds was always higher than the IAA levels found in the single flower buds. In contrast, the amounts of JA and GA in single flower buds were significantly higher as compared to those in double flower buds. The amounts of all the hormones, except for GA, were higher in the female flowers as compared to the male flowers.

We mapped DEGs to the genes related to hormone biosynthesis and the signaling pathway of *S. sagittifolia*, and the genes related to hormone synthesis and metabolism exhibited varied patterns of expression in different tissues. The number of DEGs involved in the biosynthesis and signal transduction of IAA was significantly greater than those of other types of hormone metabolism. The level of expression of AUX/IAA transcription regulation family genes related to IAA signals was affected by the expression of AUX biosynthesis and signal transduction related genes, such as *YUC*, *ARR*, *PIN*, *ARF*2, and their orthologous genes. In the female and male flowers of the two varieties, the level of expression of most IAA hormone-related DEGs significantly increased with the development of flowers (Figure 7B).

The level of expression of *LOX*, *OPR11*, *AOC4*, *MYC*, and their orthologous genes related to JA signaling and responses significantly decreased with the development of flowers (Figure 7D). The genes involved in ZR synthesis and signal transduction, such as *LOG*, *IPT*, *LOG*, *ARR*, *CRF3*, *WOX9*, and *CLV1*, and their orthologous genes exhibited the highest level of expression in S1 of flower development of the two varieties, and the level of expression then gradually decreased (Figure 7F). The highest level of expression was measured for the genes involved in GA synthesis and signal transduction, such as *GA20*, *DELL*, *GID*, and their orthologous genes (Figure 7G–H). Most of the IAA hormone-related DEGs were expressed at higher levels in double flowers, while most of the DEGs related to GA and JA hormone synthesis and metabolism were expressed significantly higher in single flowers. The results of hormone-related DEG expression are consistent with the results of the determination of endogenous hormones in flower buds, which further verifies these experimental results.

### 2.8. Functional Validation of SsAP2

We found that there was a high sequence similarity between a candidate gene (transcript_135193) and *AP2*. Interestingly, the other gene (transcript_123722) was nearly the same as the first one, but the miR172 binding site was missing in the C-terminus (Supplementary Table S8). AP2 protein combined with two predicted DNA binding domains, and it encoded a hypothetical full-length protein of 523 amino acids. The expression of *SsAP2* increased during the early three stages of flower development, and the expression of *SsAP2* in double flowers was significantly higher than that in single flowers.

**Figure 8:**
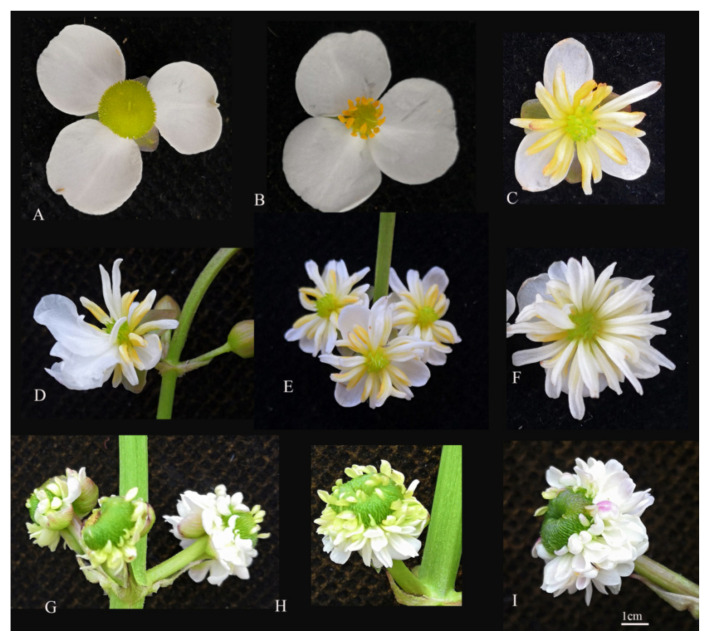
Overexpression of the *SsAP2* (*Cg123722*) transgene leads to abnormal floral organs in *S. sagittifolia*. Scale bar = 1 cm. (**A**) Control: wild-type and empty vector transgenic plants exhibit the same normal flowers: wild-type *S. sagittifolia* female flower. (**B**) Wild-type *S. sagittifolia* male flower with normal sepals, petals, carpels, or stamens. (**C**) A 35S::*SsAP2* transgenic male flower with 3 normal petals, and 6–8 petalized stamens. (**D**–**F**) A 35S::*SsAP2* transgenic male flower with 7–25 petalized stamens. (**G**) A 35S::*SsAP2* transgenic female flower with smaller petals, 16–20 transitional petals, and partial carpels. (**H**,**I**) A 35S::*SsAP2* transgenic female flower with smaller petals, 55–75 petals, and partial carpels.

To provide additional information to verify the function of this gene, we conducted a study of transgenic plants. The construction of the transgenic vector and the transformation are described in Supplementary Table S9. In this study, a total of 18 35S::*SsAP2* transgenes in single flowers was obtained by *Agrobacterium tumefaciens*-mediated inflorescence infection. No abnormal phenotype was observed in 2 plants, and there was an obvious transgenic phenotype for the other 16 plants. The overexpression of the *SsAP2* (*Cg123722*) transgene led to abnormal floral organs in *S. sagittifolia* (Figure 8). The male flower of the 35S::*SsAP2* transgene with petalized stamens contained three normal petals and 6–25 petalized stamens (Figure 8C–F). In the female flowers of the 35S::*SsAP2* transgenic *S. sagittifolia*, the carpels changed into transitional petals (16–75) (Figure 8G–I).

**Figure 9:**
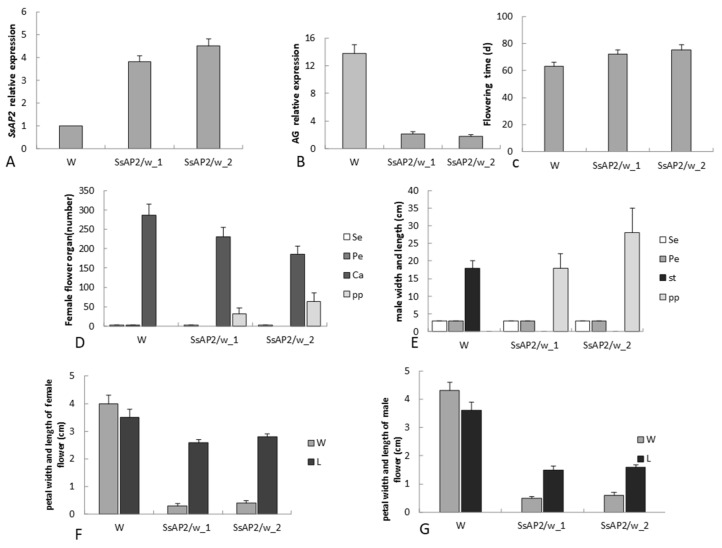
The differences between wild-type flowers and transgenic flowers. (**A**,**B**) *SsAP2* and *AG* expression in transgenic *S. sagittifolia*. (**C**) Flowering time difference between wild-type and transgenic *S. sagittifolia.* (**D**,**E**) The number of floral organ differences between wild-type and transgenic *S. sagittifolia* in female and male flowers. (**F**,**G**) Width and length differences in petals or petalized stamens and carpels of wild-type and transgenic *S. sagittifolia* in female and male flowers. Sepal (Se), petal (Pe), stamen (St), and petalized stamens (pp). Notes: W denotes the wild-type plant; *SsAP2*/w_1 denotes a 35S::*SsAP2* transgenic wild-type with a weak phenotype, and *SsAP2*/w_2 denotes a 35S::*SsAP2* transgenic wild-type with a strong phenotype.

The overexpression of transgenic *S. sagittifolia* was perpetuated for two consecutive generations, which showed that its phenotype was stable. To study the relationship between gene expression and the phenotype of transgenic *Sagittaria* in greater detail, we examined the expression of the *SsAP2* gene in 35S::*SsAP2* transgenic *Sagittaria* plants. The expression of the *SsAP2* gene in 35S::*SsAP2* transgenic plants with a strong phenotype was significantly higher than that in the transgenic plants without the phenotype and negatively correlated with the expression of *AG* (Figure 9A,B). Among the transgenic *Sagittaria* with a strong phenotype, there was no significant difference in the expression of the *SsAP2* gene, which indicated that the phenotypic difference between them was not caused by their own expression of the *SsAP2* gene. The flowering time of the transgenic plants was approximately 10 days later than that of wild plants (Figure 9C). The number of petals increased in the transgenic plants (Figure 9D,E), but the petals were significantly smaller than those observed in the wild-type (Figure 9F,G).

## 3. Discussion

### 3.1. SMRT and Illumina RNA-Seq Provided Full Length Transcriptome Information for S. sagittifolia

In recent years, third-generation transcriptome sequencing platforms such as PacBio, Nanopore, and Moleculo have become a better choice for obtaining full-length transcripts. They provide an opportunity for further study of many molecular mechanisms such as development and metabolism [28,29]. The genome of *S. sagittifolia* has not yet been published, and though transcriptome sequencing has been reported on bulb development, little is known regarding its flower transcriptome [30]. To the best of our knowledge, this study is the first to use SMRT combined with RNA-Seq sequencing technology to analyze the full-length transcriptome of *S. sagittifolia*. These transcripts will be valuable resources for various gene structures and sequences and can be directly used for genetic function research without additional gene cloning.

### 3.2. Differentially Expressed Transcripts Reveal Genes Related to the Formation and Development of Double Flowers

Double flowers are the most important ornamental feature of *S. sagittifolia*. The MADS-box plays a key role in the development of double flowers [31,32]. In this study, 103 MADS genes related to flower development were identified by further comparative analysis of the transcriptome libraries of single and double female and male flowers of *S. sagittifolia* at different developmental stages. Most of the DEGs were obtained in the S1-vs-S3 stage, which was in accordance with the results from published reports on other species, such as wild apple (*Malus sieversii*) [33], common camellia (*Camellia japonica*) [34], sacred lotus (*Nelumbo nucifera*), and tree peony (*Paeonia suffruticosa*) [35]. In these studies, many differentially expressed genes related to flower development were detected at the early stage of flower development. Most genes were downregulated in the double flower compared with the single flower of *S. sagittifolia*. There are similar reports on double peach flowers (*Prunus persica*) and double carnations (*Dianthus caryophyllus*), indicating that the formation of double flowers is negatively regulated by most genes. To understand the differential genes related to the formation of double flowers in more detail, we used WGCNA to construct a differential gene co-expression network. Among the 22 modules identified, five co-expression modules were highly correlated with the development of *S. sagittifolia* flowers. The functional annotations of these highly related module genes indicate mainly AP2, WRKY, bHLH, and MADS-box TFs, and plant hormone signal-related TFs.

TFs are a key component of the transcriptional regulation mechanism, which plays an important role in plant growth and development by regulating gene expression [27,36]. We found 5980 TFs from 67 families in the transcription, and most of the transcripts were members of the bHLH, MYB, NAC, B3, C_2_H_2_, bZIP, AP2/ERF-ERF, MADS, and WRKY families. These factors are widely involved in plant growth and are related to the formation of flowers. WGCNA confirmed that the functions of the TFs from each module were mainly concentrated in endoplasmic reticulum protein processing, splicing, plant hormone signal transduction, and starch and sucrose metabolism. Thus, in addition to the MADS-box gene related to flower development, TFs related to plant hormone signal regulation play an important role in the development of double flowers.

These TFs can provide a reference for the further study of *S. sagittifolia* flower development and double flower formation. The results of sequencing and data analysis laid a foundation for further study of genes related to the development and formation of double flowers. The DEGs and TFs identified in this study provide genetic resources for the further analysis of the function of flowering-related genes and the molecular mechanism of double flower formation.

### 3.3. Comparative Analysis the Expression of DEGs Related to Hormone Synthesis and Signal Metabolism in Single Double Flowers of S. sagittifolia

Research on plant endogenous hormones is currently focused on the responses to abiotic stress. The roles of plant seeds, roots, stems, leaves, flowers, and colors have been extensively reported, but there are few reports on the formation of double flowers [26,37]. For the first time, we compared the dynamic changes in endogenous hormones that occur during the development of flower buds of single and double flowers in *S. sagittifolia*, and many DEGs related to biosynthesis and signal transduction of IAA, JA, ZR, and GA were identified. It was reported that the expression of AUX/IAA transcriptional regulatory genes related to IAA was affected by AUX biosynthesis and signal transduction genes. Auxin and response factors act as transcriptional factors to regulate the expression of auxin response genes by binding to specific sequences of auxin response elements, and they play a role in flower development through transcriptional activation or inhibition [38,39].

In the current study, the expression levels of *IAA8*, *ARF6*, and *YUC* related to IAA biosynthesis and signal transduction were significantly higher in double flowers than those in single flowers, and those in female flowers was significantly higher than those in male flowers. However, how *AUX* response factors interact with each other in *S. sagittifolia* to affect the formation of double flowers are still unclear. JA is primarily involved in the regulation of late development of flower organs in Arabidopsis, such as the maturation of stamens and the size of petals, and exogenous application can partially restore the phenotype of petals and stamens in mutants [40]. JA affects the normal development of floral organs by affecting the expression of *LOX* and *MYC* related to signal transmission and response, resulting in an abnormal double value phenotype [12,41]. In this study, *LOX*, *OPR11*, *AOC4*, and *MYC*, and their homologous genes related to JA signal transduction and response in double flowers were significantly lower than those in single flowers. It has been reported that JA can also interact with IAA to regulate the development of flower organs, including the development of stamens and the fertility of pollen. Therefore, during the process of flower development, the molecular mechanism and interaction mechanism of various plant hormones that regulate the development of organs in *S. sagittifolia* double flowers merit further study.

### 3.4. C-Function AG Gene Associated with Double Flower Formation

Double flowers are characterized by an increase in petals or petal area, which is of substantial ornamental value. Many research results on various ornamental plants show that the occurrence of several double flowers is closely related to the change in C functions [10,42]. In *A. thaliana*, C-type genes determine the characteristics of expression of stamens and carpels and participate in the decisive control of inflorescences. A mutation in the C-function *AG* gene leads to the homologous transformation from stamens to petals, and the number of stamens decreases, while the number of petals increases [43]. Currently, this has been similarly reported in many species, including Japanese morning glory (*Pharbitis nil*) [44], Japanese mustard [45], common stock (*Matthiola incana*) [10], and Japanese rose (*Rosa rugosa* Thunb) [8,12]. The loss of function or abnormal expression of the C homologous gene often leads to the formation of double flowers.

In the current study, the morphological characteristics of the double-flowered *S. sagittifolia* flower during different developmental stages were studied using paraffin sections. Similar to the single flower, the double flower has normal sepals and petals in the first and second whorls. However, the stamens and carpels are missing, and there is an increased number of petals in the third whorls. The morphological features of double flowers are diverse in shape. For example, five major different flower types have been identified in camellia [13], which shows that the molecular mechanisms that control the development of double flowers are different. Studies on double-flowered rhododendron showed that the double-flowered phenotype was caused by abnormal mRNA of the *AG*/*PLE* homologous gene in the third and fourth whorls [42]. In ‘double white’ *T. thalictroides*, the molecular analysis of the *AG ortholog ThtAG1* showed that the insertion of a retrotransposon led to the alternative splicing of a mutant protein with a K-box deletion [11]. It was similarly reported that the expression of *GsAG1* in a double-flowered mutant of *G. scabra* was downregulated because there was an insertion into the intron of the *GsAG1* gene [46,47].

In this study, *AG* and its homologous genes screened from full-length sequencing combined with RNA-seq showed complete MADS and K-box domains. In contrast to the reports on *Rhododendron* and *Gentiana rigescens*, the sequence structure of mRNA and the deduced amino acid protein function of *AG* in *S. sagittifolia* were normal. Further study on the expression pattern of *AG* and its homologous genes showed that *AG* was primarily expressed in the stamens and carpels, which indicated that *AG* was highly conservative in maintaining the third whorl stamens and the fourth whorl carpels. This was consistent with those reported in *A. thaliana*, *P. persica*, and *R. rugosa* [48,49,50]. We performed real-time quantitative PCR to compare the expression patterns of *AG* in single and double flowers of *S. sagittifolia*.

In the double flower, the expression of *AG* was significantly lower than that in single flowers during the three stages of flower development, and it was highly expressed in stamens and carpels but with little expression in sepals and petals (Figure 6C). In the current study, the phenotype of *S. sagittifolia* exhibited a complete loss of stamens and carpels and a decrease in *AG* expression. According to the ABC model gene of flower development, the downregulation of *AG* homologous gene expression leads to the homologous transformation from stamens to petals and the formation of double flowers. There have been similar findings for the rose [32], ranunculids [11], and easter lily (*Lilium longiflorum*) [51], where a decrease in *AG* expression in the third whorl leads to the homologous transformation from stamens to petals and the formation of double flowers [9].

### 3.5. Function of SsAP2 in S. sagittifolia

Many studies have shown that *AP2* and other members of its family also play an important role in the development and formation of flowers and assume an antagonistic role in regulating the expression of *AG* [15,52,53]. In the current study, we identified a gene in the euAP2 family (*SsAP2*) that was missing the C-terminal fragment, resulting in the loss of miR172 target sites. The expression of this gene was negatively correlated with *AG* expression, and the transgenic results showed that overexpression of *SsAP2* would lead to an increase in the number of petals, which indicated that *AP2* lacking the binding site of miR172 plays an important role in the formation of double flowers. This is consistent with the results of the following reports. In *A. thaliana*, overexpression of the miR172-resistant *AP2* gene leads to extra petals and an uncertain floral meristem. *AP2* antagonizes the transcriptional activity of *AG*, thus maintaining the center of the floral meristem [54,55]. In the rose genome, a TE insertion in *RcAP2L* was found to produce a new splice acceptor site, which resulted in the deletion of exons 9 and 10 and the formation of a truncated protein [8,15,53]. In *P. persica*, a similar mechanism was found to be related to the formation of double flowers, i.e., the deletion of the target site of miR172 in the candidate gene that encodes the *euAP2* transcription factor [18].

Compared with that in the necklace-shaped dendrobium (*Dendrobium moniliforme*), the mutation of the miR172 cleavage site of *DcAP2L* in *D. moniliforme* resulted in the high expression of *DcAP2L*. *DcAP2L* can continuously inhibit the expression of the *AG* gene, which finally leads to the formation of double flowers. The difference between *DcAP2L* and *dcAP2l* in bamboo (*Phyllostachys praecox*) was due to a single nucleotide polymorphism at the miR172 target site [2]. We hypothesized that the lack of the miR172 target *SsAP2* in *S. sagittifolia* reduced the degradation of *AP2*, which led to the high expression of *AP2*, antagonized the expression of *AG*, and limited the expression of *AG* in the flower center. This may play a positive role in promoting *WUS* in the floral meristem and affect the *WUS-AG* feedback regulatory loop. This will eventually lead to the homologous transformation of stamens and carpels to petals, and the increase in flower organs will result in the formation of a double flower. The specific mechanism of this regulation is worthy of further study.

In the current study, the lack of *SsAP2* overexpression by miR172 also led to a delay in flowering time and an increase in petals, but the petals were smaller (Figure 9). Combined with the WGCNA module analysis, it was found that transcription factors regulated by plant hormone synthesis (especially IAA) and signal pathway metabolism signals played an important role in the development of double flowers. It was speculated that in addition to *SsAP2*, IAA may be involved in regulating the formation of double flowers, but the specific interactions are unclear and merit further study. However, transgenic *S. sagittifolia* only formed semi-double petals, and the inner third-whorl stamens and inner third-whorl carpels still existed (Figure 8). This indicates that in addition to the role of *SsAP2*, the formation and development of double flowers may also be affected by other regulatory factors, and the specific mechanism of regulation merits further study.

## 4. Materials and Methods

### 4.1. Plant Materials

The experimental materials included single-flowered (*S. sagittifolia* var. *wu cig*) and double-flowered (*S. sagittifolia* var. *Flore Pleno*) *S. sagittifolia*. The plants were obtained from the experimental base of the Guangxi Academy of Agricultural Sciences, Guangxi Zhuang Autonomous region, China (2283.15′–2283.67′ N, 10,831.64′–10,937.45′ E). Female and male flower buds of *S. sagittifolia* were collected according to the description in Supplementary Figure S1, from June to August in 2019 between 4:00 and 8:00 a.m., and at least 20 flower buds from each sample were mixed. After washing with sterile double distilled water, they were quickly frozen in liquid nitrogen, stored at −80 °C, and then sent to Nuohe Zhiyuan Technology Co., Ltd. (Beijing, China) for sequencing.

All the samples were named according to the abbreviation for variety name and developmental period, e.g., the abbreviations for single female and male flowers were SC and SX, respectively, whereas the abbreviations for double female and male flowers were DC and DX, respectively. The abbreviations for flower bud differentiation in the three stages were S1, S2, and S3. Female and male flower buds were collected at three different developmental stages for single and double flowers (Supplementary Table S1). The number of samples of single flower buds in S1 was insufficient, and thus, there were only two biological replicates, while the others included three biological replicates. A total of 34 tissue samples were subjected to RNA-Seq.

### 4.2. Preparation and Observation of Paraffin Sections

Sampling was initiated from the time of drawing of the flower buds. After washing with distilled water, a sample was fixed in FAA (formalin: glacial acetic acid: 50% ethanol = 8:5:87 [*v*/*v*/*v*]) for 24 h. The fixed materials were dehydrated with an ethanol gradient, made transparent with xylene, soaked in wax and embedded, and then sliced to form pieces that were 8–10 mm thick. The slices were stained using a saffron-solid green staining method and then observed and photographed using a Nikon-YS100 microscope (Nikon, Tokyo, Japan).

### 4.3. Determination of the Content of Endogenous Hormones

A standard solution was prepared by accurately weighing 1 g of *S. sagittifolia* flower bud samples, grinding in liquid nitrogen, extracting them twice at 4 °C, and mixing the supernatants. The extract was purified using a C18 solid phase extraction column and filtered through a 0.2 μm organic microporous membrane. The amount of light was minimized during the operation, and the temperature remained at < 4 °C. The chromatographic conditions were as follows: Agilent C18 column (Agilent Technologies, Santa Clara, CA, USA) (2.1 mm × 50 mm, 1.8 μm); column temperature of 30 °C; sample injection volume of 2 μL; and a flow rate of 0.2 mL min^−1^. Mobile phase A was HPLC grade methanol, and B was 1 mmol L-1 glacial acetic acid solution. The conditions for mass spectrometry were as follows: electrospray ion source (IAA, ZR-positive ion detection; JA, GA3 negative ion detection); multi-reaction monitoring scan; ion spray voltage of 3000 V; atomizing gas pressure of 275 kPa; sheath flow gas temperature of 350 °C; velocity of sheath flow gas at 10 mL min; capillary voltage of 3500 V; ion source temperature of 325 °C; and a flow rate of desolvation gas at 8.0l min^−1^. The extraction method utilized the instrument parameters and method of Netal [56].

### 4.4. RNA Extraction and Library Construction

The method of Liu et al. [57] was used to obtain the total RNA of *S. sagittifolia* flower buds at different developmental stages. The integrity of the total RNA samples was monitored by the Agilent 2100 system, and the purity of the RNA samples was measured using NanoDrop spectroscopy (Novogene Company, Beijing, China). The mRNA was isolated from the mixed RNA sample by magnetic beads with oligo (dT) and then reverse transcribed into cDNA using a SMARTer cDNA synthesis kit (Takara Biotechnology, Dalian, China). The cDNA was then screened using BluePippin (Sage Science, Beverly, MA, USA), and the full-length cDNA was repaired. It was then connected using a smart dumbbell connector. Finally, the library was obtained by digestion with exonuclease. After the library was successfully constructed, the full-length transcriptome was sequenced using the PacBio Sequel™ sequencing platform.

### 4.5. Quality Control of the Full-Length Transcriptome and UniGene Library

SMRT analysis software was used to analyze the original data by filtering, classifying, and ice (iterative iso form-clustering) clustering correction under default parameters. The full-length non-coding (FLNC) sequence was subsequently obtained by classification. The FLNC sequences were clustered by CD-HIT software to obtain the UniGene library, and gene function annotation and structural analysis were then carried out.

### 4.6. Annotation of Sequence Information

Using the BLASTx tool, combined with the Nr, Swiss-Prot, KEGG, and COG/KOG protein databases, the protein sequence with the highest similarity to the isoform sequence was obtained. This enabled the protein annotation information for the isoforms to be obtained. Based on the basic function annotation information, protein function annotation, pathway annotation, COG/KOG function annotation, and Gene Ontology (GO) functional annotation were conducted, among others.

### 4.7. Other Structural Analyses

Other structural analyses were performed, including the coding sequence (CDS) prediction, which involved a comparison of the isoforms with Nr, Swiss-Prot, KEGG, COG/KOG, and other protein libraries (e value < e^−5^). The most highly similar protein in the comparison results was used as the reference for the coding region sequence of the isoform and translated based on the standard codon table to obtain the nucleic acid and amino acid sequences of the coding region. Finally, the coding region of an isoform that did not match the protein library above was predicted using ANGEL [58], and the nucleic acid and amino acid sequences of the coding region were obtained.

*Protein domain analysis*: The Pfam database (version 26.0) was used for comparison, and the annotation information related to egg white structure encoded by the isoform was obtained. Simultaneous hmmer-profile hidden Markov models for biological sequence analysis (https://www.ebi.ac.uk/Tools/hmmer/, accessed on date 24 May 2021) were used to predict the protein sequence encoded by the isoform. It was then compared with the SMART database (version number SMART 06/08/2012), and the annotation information related to the protein structure encoded by the isoform was obtained.

*Transcription factor prediction*: The predicted protein sequence was compared with the corresponding transcription factor (TF) database (Plant TFdb) by hmmscan to obtain the predicted TF type and quantity. SSR: SSR in the full-length transcriptome was searched using the software MISA [59]. LncRNA and AS: Coding Non-Coding Index software (CNCI) [60] and CPC software [61] were used to predict the coding ability. The results predicted by both software packages as ‘non-coding’ were used as the final long non-coding (lnc)RNA result for the full-length transcriptome. The coding sequences were assembled using the software Cogent [62], and variable shear analysis was then performed by SUPPA [57] as a reference.

### 4.8. Verification of Differentially Expressed Genes (DEGs)

The DEGs were selected according to the high expression and with differences expression at different developmental stages. Based on the clustering results of differentially expressed genes (DEGs), differentially expressed MADS gene primers (Table S1) were selected. Quantitative real-time reverse transcription (qRT-PCR) verification analysis was conducted with reverse transcription using the cDNA of the sequenced samples as the template.

### 4.9. Construction of the Transformation Vector and Acquisition of Transgenic Plants

Single flowers of *S. sagittifolia*, binary plasmid pBI121, TOP10 *Escherichia coli* competent cells, and *Agrobacterium tumefaciens* EHA105 were preserved in our laboratory. The plasmid pBI121 and the target gene fragment were digested by double enzymes. Detection by electrophoresis and recovery of the *Xba*I and *Xma*I enzymatic digestion products were performed according to the manufacturer’s instructions (Sangon Biotech, Shanghai, China). The vector was ligated with the target gene, and the ligated product was transformed into TOP10 competent *E. coli* cells.

After that, a positive monoclonal plaque was singled out and cultured, and several colonies were randomly selected for PCR verification and PCR identification using universal primers. The positive clones were confirmed by sequencing, which was performed by Wuhan Gene Biological Engineering Co., Ltd. (Wuhan, China). The *SsAP2* gene expression vector was transferred into *A. tumefaciens* strain EHA105 by liquid nitrogen freezing and thawing. The EHA105 competent cells of *A. tumefaciens* were prepared as previously described, and the positive clones were identified by PCR.

### 4.10. Floral Dip of the Inflorescence of S. sagittifolia

After sprouting, the plant was incubated at 28 °C, a light intensity of 10,000 Lux, and photoperiod of 16 h (light)/8 h (dark). The plant was subjected to conventional water and fertilizer management [63]. To promote the growth of side branches, the main flower branch was severed when the plant blossomed for the first time. Buffer was prepared, and the collected bacterial strains were precipitated and suspended in the buffer and evenly mixed. The inflorescences of *S. sagittifolia* were immersed in *A. tumefaciens* solution for 90 s. Finally, the infected plants were wrapped in black plastic film. The film was opened 48 h later and it was transformed three times every 7 days to increase the rate of transformation. The mature seeds of *S. sagittifolia* were harvested after cultivation for a period of time.

### 4.11. Identification of Transgenic S. sagittifolia

The seeds of transgenic *S. sagittifolia* were identified after culturing on agar plates containing kanamycin (Kan) antibiotic. Then, the seeds were surface-sterilized in 70% ethanol for 1 min, followed by 10 min in 0.1% (*w*/*v*) mercuric chloride and three rinses with sterile distilled water. For 7 days, they were pretreated with water at a low temperature (4 °C), and the seeds were germinated on ½ Murashige and Skoog (MS) media that contained 50 mg/L of Kan at 25 °C and 12 h/12 h (light: dark). The seedlings were cultured under continuous light for 7–10 days.

After the transgenic seeds germinated on MS + Kan plates and grew for 2 weeks, the plants determined as positive by PCR were transferred into soil for further growth. The plants that could germinate and grow normally in the presence of antibiotics were transgenic-positive plants. Primers for constructing a transgenic expression vector were used to identify the positive transgenic seedlings by PCR.

## 5. Conclusions

We conducted the first transcriptome analysis of the flower of *S. sagittifolia* that utilized the combination of SMRT and Illumina RNA-Seq. Many MADS-box genes related to the formation of double flowers were obtained. WGCNA showed that plant hormone signal-regulated transcription factors played an important role in the development of double flowers. We also identified an *AP2* orthologous gene, *SsAP2*, with a deletion at the binding site for miR172, as the expression of *AP2* negatively correlates with the expression of *AG*, this may play a positive role in the promotion of *WUS* in the floral meristem and affect the *WUS-AG* feedback regulation loop, which may lead to an increased number of petals and form double flowers. To the best of our knowledge, this is the first report of a full-length transcriptome of *S. sagittifolia*. These reference transcripts will be valuable resources for the analysis of gene structures and sequences, and provide a theoretical basis for the molecular regulatory mechanism of the formation of double flowers. They are of substantial theoretical and practical significance for breeding additional new varieties and using innovative germplasm to develop high-quality double flowers in the future.

## Data Availability

The datasets generated for this study can be found as K5F75 (SAMN20841752) in the GenBank database.

## Supplementary Materials

The following supporting information can be downloaded at: https://www.mdpi.com/article/10.3390/ijms23042240/s1.
