# Supplementary material for: SMRT and Illumina RNA-Seq Identifies Potential Candidate Genes Related to the Double Flower Phenotype and Unveils SsAP2 as a Key Regulator of the Double-Flower Trait in Sagittaria sagittifolia
Source: Int J Mol Sci. 2022 Feb 17;23(4):2240. doi: 10.3390/ijms23042240 (PMC8875719; doi:10.3390/ijms23042240)
Supplement: Supplementary file 1 [file ijms-23-02240-s001.zip › Supplementary Table 1-Primers used for differential gene expression.pdf]

Table 1 genes Primer of *Sagittaria sagittifolia*

14296（AP2）	
14926F	5’ AAACCGCAATAGCGACCG 3’
14926R	5’ GGCTGCCAGAACCAATACCTC 3’
12921R	5’ TGCCAGAACCAATACCTCCA 3’
42875（AP3）	
42875F	5’ AGACAGAAGCACATCCCCACC 3’
42875R	5’ CGATCTTGCCACGACCCAT 3’
42092（AG）	
42092F	5’ ACCTTAACCAACCCCTCCACT 3’
42092R	5’ CCGTTCAACGATCCTCTGC 3’
49250（SEP）	
49250F	5’ AGACAGAAAAGAGTTGGATGCG 3’
49250R	5’ CGAGGGGTAAGTGAGATAGGCT 3’
48048（PI）	
48048F	5’ CCCTCACCACCACTCCTTTC 3’
48048R	5’ CAATCTCCCAGCAGCGTCA 3’
140412（WUS）	
140412F	5’ ATCTTGGATTATGCCTGTGCC 3’
1404R	5’ CATCATGGACGTTTGACTGACC 3’
SsAP2-F	5’ TCTAGAATGCAGCAGTGAGGCCGGAGGTCAGG3’
SsAP2-R	5’TCTAGAATGCAGCAGTGAGGCCGGAGGTCAGGACAC3’
