# Supplementary material for: SMRT and Illumina RNA-Seq Identifies Potential Candidate Genes Related to the Double Flower Phenotype and Unveils SsAP2 as a Key Regulator of the Double-Flower Trait in Sagittaria sagittifolia
Source: Int J Mol Sci. 2022 Feb 17;23(4):2240. doi: 10.3390/ijms23042240 (PMC8875719; doi:10.3390/ijms23042240)
Supplement: Supplementary file 1 [file ijms-23-02240-s001.zip › Supplementary Table 2-Statistical analysis of sample sequencing data.pdf]

Supplementary Table 2-1. RNA-Seq statistical of sample sequencing data evaluation of *Sagittaria sagittifolia*

Samples	**Read Number**	**Base Number**	**GC Content**	**%≥Q30**
SC2-1	31,529,264	9,429,799,036	53.14	94.32
SC2-2	22,284,298	6,651,291,022	53.48	94.24
SC2-3	21,622,935	6,446,993,312	53.78	94.15
SX2-1	24,017,219	7,182,145,440	53.15	94.67
SX2-2	39,127,198	11,670,597,042	53.79	94.16
SX2-3	22,242,407	6,632,086,748	53.52	93.95
DC3-1	23,544,845	7,021,875,568	53.36	94.34
DC3-2	21,169,992	6,310,914,946	53.19	94.23
DC3-3	26,127,702	7,796,294,954	53.40	94.82
DC2-1	23,127,300	6,912,360,434	52.32	94.39
DC2-2	21,620,720	6,457,177,220	52.39	94.79
DC2-3	22,231,989	6,634,423,992	52.99	94.53
DC1-1	20,125,975	6,009,580,162	52.68	94.46
DC1-2	19,648,755	5,878,892,078	52.45	94.04
DC1-3	24,508,029	7,326,406,122	52.87	94.58
SC3-1	20,628,133	6,154,150,226	53.29	94.48
SC3-2	23,152,671	6,879,804,752	53.27	93.97
SC3-3	25,581,597	7,622,873,756	53.64	94.48
SX3-1	21,867,569	6,533,215,600	53.93	94.92

SX3-2	25,198,833	7,492,023,170	53.83	94.21
SX3-3	27,311,783	8,139,566,390	53.69	94.54
SC1-1	22,131,161	6,590,590,316	52.04	94.84
SC1-2	22,849,152	6,816,839,492	52.17	94.61
SX1-1	22,002,282	6,573,979,094	52.29	94.39
SX1-2	22,775,623	6,801,539,948	52.56	94.65
DX3-1	19,897,979	5,951,012,156	52.45	95.27
DX3-2	29,174,702	8,696,540,448	53.41	94.63
DX3-3	21,329,167	6,353,392,404	53.36	94.39
DX2-1	24,057,177	7,191,361,348	52.56	94.90
DX2-2	24,175,413	7,216,120,786	53.27	94.35
DX2-3	20,835,011	6,208,674,078	52.88	95.13
DX1-1	19,823,039	5,923,366,726	52.48	94.30
DX1-2	21,756,889	6,502,916,340	52.66	94.50
DX1-3	21,815,010	6,518,364,440	51.82	94.97

note：Samples：Sample information list Sample name；

Read Number：Clean Data in total number of pair-end Reads；

Base Number：Clean Data in total base number；

GC Content：Clean Data GC Content， Clean Data G and C in percentage of the total base；

%≥Q30：Clean Data with the percentage of a base with mass values greater than or equal to 30.

Supplementary Table2- 1 Categorize information of transcripts in *Sagittaria sagittifolia* from SMRT data

Classify	Numbers
Number of ccs	656,275
Read Bases of CCS	1,466,214,311
Mean Read Length of CCS	2,234
Number of undesired primer reads	50,432
Number of filtered short reads	57

Number of full-length non-chimeric reads	579,295
Number of consensus isoforms	156,338
Average consensus isoforms read length	2,208
Number of polished high-quality isoforms	153,620
Number of polished low-quality isoforms	2,315
Percent of polished high-quality isoforms(%)	98.26%

Supplementary Table 2- 3 Statistics of transcripts noted

**Annotated databases**	**Isoform Number**
COG	24,381
GO	42,556
KEGG	25,831
KOG	38,191
Pfam	47,759
Swiss-Prot	43,854
eggNOG	58,350
nr	59,388
All	59,789
