# Supplementary material for: SMRT and Illumina RNA-Seq Identifies Potential Candidate Genes Related to the Double Flower Phenotype and Unveils SsAP2 as a Key Regulator of the Double-Flower Trait in Sagittaria sagittifolia
Source: Int J Mol Sci. 2022 Feb 17;23(4):2240. doi: 10.3390/ijms23042240 (PMC8875719; doi:10.3390/ijms23042240)
Supplement: Supplementary file 1 [file ijms-23-02240-s001.zip › Supplementary Table 4-. A comparison of second-generation sequencing data with third-generation non-redundant transcript statistics.pdf]

Supplementary Table 4 Statistics of data filtering from RNA-Seq data of *S. sagittifolia*

Samples	Total Reads	Uniquely mapped reads %	% of reads mapped to multiple loci	% of reads mapped to too many loci
SC2-1	31,529,264	24.31%	59.51%	4.62%
SC2-2	22,284,298	23.81%	58.49%	5.08%
SC2-3	21,622,935	24.32%	58.36%	4.94%
SX2-1	24,017,219	24.62%	58.75%	4.07%
SX2-2	39,127,198	24.37%	57.26%	4.40%
SX2-3	22,242,407	24.11%	57.17%	4.43%
DC3-1	23,544,845	23.35%	60.64%	4.63%
DC3-2	21,169,992	22.49%	60.26%	5.24%
DC3-3	26,127,702	23.28%	60.78%	4.79%
DC2-1	23,127,300	24.63%	60.19%	4.64%
DC2-2	21,620,720	23.03%	57.74%	7.65%
DC2-3	22,231,989	25.30%	59.92%	4.55%
DC1-1	20,125,975	24.04%	57.29%	7.73%
DC1-2	19,648,755	25.13%	56.66%	7.12%
DC1-3	24,508,029	25.10%	58.34%	6.43%
SC3-1	20,628,133	21.49%	56.19%	4.31%
SC3-2	23,152,671	22.02%	55.07%	3.85%
SC3-3	25,581,597	21.79%	55.53%	4.09%
SX3-1	21,867,569	23.58%	53.82%	3.40%
SX3-2	25,198,833	22.88%	54.01%	3.84%
SX3-3	27,311,783	23.12%	53.15%	3.80%
SC1-1	22,131,161	21.95%	52.62%	4.66%
SC1-2	22,849,152	23.22%	52.91%	4.12%
SX1-1	22,002,282	22.69%	57.55%	5.95%
SX1-2	22,775,623	22.77%	55.34%	5.44%
DX3-1	19,897,979	22.74%	63.26%	4.43%
DX3-2	29,174,702	22.12%	61.87%	4.92%
DX3-3	21,329,167	23.04%	60.99%	4.50%
DX2-1	24,057,177	23.82%	60.74%	4.30%
DX2-2	24,175,413	24.05%	59.75%	4.31%
DX2-3	20,835,011	23.82%	60.16%	4.39%
DX1-1	19,823,039	25.46%	57.29%	5.08%
DX1-2	21,756,889	25.31%	59.01%	5.36%
DX1-3	21,815,010	24.88%	59.73%	5.18%
