# Supplementary material for: SMRT and Illumina RNA-Seq Identifies Potential Candidate Genes Related to the Double Flower Phenotype and Unveils SsAP2 as a Key Regulator of the Double-Flower Trait in Sagittaria sagittifolia
Source: Int J Mol Sci. 2022 Feb 17;23(4):2240. doi: 10.3390/ijms23042240 (PMC8875719; doi:10.3390/ijms23042240)
Supplement: Supplementary file 1 [file ijms-23-02240-s001.zip › Supplementary Table 8-Two AP2 homologous gene sequence alignments and description.pdf]

A) CDS sequence alignments for *Cg1351933 and Cg123722*

```
               10        20        30        40        50        60
           ....|....| ....|....| ....|....| ....|....| ....|....| ....|....|
Cg1351933   ATGATGCTGG ATCTCACCCT GGCGTCGTCC CCTGACGACA CGTACAAGGG CAGCGTGGAA  60
Cg123722    ATGATGCTGG ATCTCACCCT GGCGTCGTCC CCTGACGACA CGTACAAGGG CAGCGTGGAA  60
Clustal Co  ********** ********** ********** ********** ********** **********  60
               70        80        90       100       110       120
           ....|....| ....|....| ....|....| ....|....| ....|....| ....|....|
Cg1351933   GCCTCCGTGA ACGAGTCCGG AACGTCCAAG TCCTCCGTCC TCAACGGAGA CGGCTCCAGC  120
Cg123722    GCCTCCGTGA ACGAGTCCGG AACGTCCAAG TCCTCCGTCC TCAACGGAGA CGGCTCCAGC  120
Clustal Co  ********** ********** ********** ********** ********** **********  120
              130       140       150       160       170       180
           ....|....| ....|....| ....|....| ....|....| ....|....| ....|....|
Cg1351933   AACGACGATT CATGCTCCAA CCGCGTTGCC GCGGCGGGGT TCCGCTTCGG CATCCTCAAG  180
Cg123722    AACGACGAGT CATGCTCCAA CCGCGTTGCC GCGGCGGGAT TCCGCTTCGG CATCCTCAAG  180
Clustal Co  ******** * ********** ********** ******** * ********** **********  178
              190       200       210       220       230       240
           ....|....| ....|....| ....|....| ....|....| ....|....| ....|....|
Cg1351933   GACGGGGACG ACGAAGATGA CGACTTGGTT GAGCAGGAGG TGCCCAACGA CGACGATGGT  240
Cg123722    GACGGGGACG ACGAAGATGA CGACTTGGTT GAGCAGGAGG TGCCCAACGA CGACGATGGT  240
Clustal Co  ********** ********** ********** ********** ********** **********  238
              250       260       270       280       290       300
           ....|....| ....|....| ....|....| ....|....| ....|....| ....|....|
Cg135193    GTTGACGATG ACGGGGCGTT TGGCCGGATG GGGGGCCTCG CCACCAAGGA CTTGTTCCCG  300
Cg123722    GTTGACGATG ACGGGGCGTT TGGCCGGATG GGGGGCCTCG CCACCAAGGA CTTGTTCCCG  300
Clustal Co  ********** ********** ********** ********** ********** **********  298
              310       320       330       340       350       360
           ....|....| ....|....| ....|....| ....|....| ....|....| ....|....|
Cg135193    CCACCTATGA TGGCCTGCGT GGAAGGCACC CTGGGAGCGA ACGCTGCGGG TATACGGGCT  360
Cg123722    CCACCTATGA TGGCCTGCGT GGAAGGCACC CTGGGAGCGA ACGCTGCGGG TATACGGGCT  360
Clustal Co  ********** ********** ********** ********** ********** **********  358
              370       380       390       400       410       420
           ....|....| ....|....| ....|....| ....|....| ....|....| ....|....|
Cg1351933   GTTCCCTTCG TTCCTGTGGG ATCTTCGTCG TCCTCTGCTG CCTGCAGGCC GCAGTTGGTG  420
Cg123722    GTTCCCTTCG TTCCTGTGGG ATCTTCGTCG TCCTCTGCTG CCTGCAGGCC GCAGTTGGTG  420
Clustal Co  ********** ********** ********** ********** ********** **********  418
              430       440       450       460       470       480
           ....|....| ....|....| ....|....| ....|....| ....|....| ....|....|
Cg135193    GATCTGAGTT TCTGCCGGAG GGATGCAGCA GTGAGGCCGG AGGTCAGGAC ACTCCCTCAG  480
Cg123722    GATCTGAGTT TCTGCCGGAG GGATGCAGCA GTGAGGCCGG AGGTCAGGAC ACTCCCTCAG  480
Clustal Co  ********** ********** ********** ********** ********** **********  478
              490       500       510       520       530       540
           ....|....| ....|....| ....|....| ....|....| ....|....| ....|....|
Cg1351933   CAGCCGCCGC CGCCACCGCC GCAGCAGCAG CAGCCGCAAC AACAGGTGAA GAAGAGCCGG  540
Cg123722    CAGCCGCCGC CGCCGCCGCC GCAGCAGCAG CABCCGCAAC ATCAGGTGAA GAGAAGCCGG  540
Clustal Co  ********** **** ***** ********** ** ******* * ******** **  ******  533
              550       560       570       580       590       600
           ....|....| ....|....| ....|....| ....|....| ....|....| ....|....|
Cg1351933   CGTGGCCCAC GGTCCCGAAG CTCGCAGTAC CGTGGCGTCA CGTTCTACCG TAGGACTGGA  600
Cg123722    CGTGGCCCAC GGTCCCGAAG CTCGCAGTAC CGTGGCGTCA CGTTCTACCG TAGGACTGGA  600
Clustal Co  ********** ********** ********** ********** ********** **********  593
              610       620       630       640       650       660
           ....|....| ....|....| ....|....| ....|....| ....|....| ....|....|
Cg135193    AGATGGGAAT CCCACATTTG GGACTGTGGA AAGCAGATCT ATCTAGGTGG CTTTGACACT  660
Cg123722    AGATGGGAAT CCCACATTTG GGACTGTGGA AAGCAGATCT ATCTAGGTGG CTTTGACACT  660
Clustal Co  ********** ********** ********** ********** ********** **********  653
              670       680       690       700       710       720
           ....|....| ....|....| ....|....| ....|....| ....|....| ....|....|
Cg135193    GCTCATGCCG CAGCAAGGGC ATATGACCGG GCTGCTATAA AATTTAGGGG AATAGATGCA  720
Cg123722    GCTCATGCCG CAGCAAGGGC ATATGACCGG GCTGCTATCA AATTTAGGGG AATAGATGCA  720
Clustal Co  ********** ********** ********** ******** * ********** **********  712
```

```
                730       740       750       760       770       780
        ....|....| ....|....| ....|....| ....|....| ....|....| ....|....|
Cg135193    GACATAAATT TCAACCTCAG TGACTACGAT GATGATATGA AACTGATGAG CAATCTCTCC  780
Cg123722  GACATAAATT TCAACCTCAG TGACTACGAT GATGATATGA AACTGATGAG CAATCTCTCC  780
Clustal Co ********** ********** ********** ********** ********** **********  772
                790       800       810       820       830       840
        ....|....| ....|....| ....|....| ....|....| ....|....| ....|....|
Cg135193    AAGGAGGAAT TCGTTCACAT ATTGCGTCGC CAAAGTACAG GATTTTCAAG AGGAAGTTCA  840
Cg123722  AAGGAGGAAT TCGTTCACAT ATTGCGTCGC CAAAGTACAG GATTTTCAAG AGGAAGTTCA  840
Clustal Co ********** ********** ********** ********** ********** **********  832

                850       860       870       880       890       900
        ....|....| ....|....| ....|....| ....|....| ....|....| ....|....|
Cg135193    AAATACAGAG GGGTGACGTT GCACAAGTGT GGTCGCTGGG AAGCTCGCAT GGGGCAGTTC  900
Cg123722  AAATACAGAG GGGTGACGTT GCACAAGTGT GGTCGCTGGG AAGCTCGCAT GGGGCAGTTC  900
Clustal Co ********** ********** ********** ********** ********** **********  892
                910       920       930       940       950       960
        ....|....| ....|....| ....|....| ....|....| ....|....| ....|....|
Cg135193    CTGGGAAAGA AGTATATATA TCTTGGATTG TTCGACAGTG AAGTAGAGGC TGCAAGGGCT  960
Cg123722  CTGGGAAAGA AGTATATATA TCTTGGATTG TGTGACAGTG AAGTAGAGGC TGCAAGGGCT  960
Clustal Co ********** ********** ********** *  ******* ********** **********  950
                970       980       990       1000      1010      1020
        ....|....| ....|....| ....|....| ....|....| ....|....| ....|....|
Cg135193    TATGACAAGG CAGCTATCCG ATGTAATGGG AGGGAGGCTG TCACAAATTT CGACCTGAGT  1020
Cg123722  TATGACAAGG CAGCTATCCG ATGTAATGGG AGGGAGGCTG TCACAAATTT CGACCTGAGT  1020
Clustal Co ********** ********** ********** ********** ********** **********  1010
                1030      1040      1050      1060      1070      1080
        ....|....| ....|....| ....|....| ....|....| ....|....| ....|....|
Cg135193    ACTTACGAAG GGGAGCTTAT CTCGGAAGCA GACACTGAGG GCCATAACCT TGACTTGAGT  1080
Cg123722  ACTTACGAAG GGGAGCTTAT CTCGGAAGCA GACACTGAGG GCCATAACCT TGACTTGAGT  1080
Clustal Co ********** ********** ********** ********** ********** **********  1070
                1090      1100      1110      1120      1130      1140
        ....|....| ....|....| ....|....| ....|....| ....|....| ....|....|
Cg135193    CTAAGTATTT CCCAGCCTGC CAGTGACAGT CCCAAGGGGG ATAAGACCCC GAATACTGTT  1140
Cg123722  CTAAGTATTT CCCAGCCTGC CAGTGACAGT CCCAAGGGGG ATAA------ ----------  1124
Clustal Co ********** ********** ********** ********** ****                1114
                1150      1160      1170      1180      1190      1200
        ....|....| ....|....| ....|....| ....|....| ....|....| ....|....|
Cg135193    CTGATCCAGT CCCTTTCATT TGACTCGCCA GAGGTTAAGA AGTCTCGGAT CGATTTTCCT  1200
Cg123722  ---------- ---------- ---------- ---------- ---------- ----------  1124
Clustal Co                                                              1114
                1210      1220      1230      1240      1250      1260
        ....|....| ....|....| ....|....| ....|....| ....|....| ....|....|
Cg135193    TCGTCCTTTT CCATCAGCCA GACATGCAGA CCAGCTGAAG GACGACACGG GCTTCCCATG  1260
Cg123722  ---------- ---------- ---------- ---------- ---------- ----------  1124
Clustal Co                                                              1114
                1270      1280      1290      1300      1310      1320
        ....|....| ....|....| ....|....| ....|....| ....|....| ....|....|
Cg135193    TGGGTGACTC AGGCCAATTG TTTTCCAAGC TTTGAGGGGA GCGCACTTGA CAAGAGGCCA  1320
Cg123722  ---------- ---------- ---------- ---------- ---------- ----------  1124
Clustal Co                                                              1114
                1330      1340      1350      1360      1370      1380
        ....|....| ....|....| ....|....| ....|....| ....|....| ....|....|
Cg135193    GAGACGAAAC CGATTGCTCT TCCAAGTTTG TCATGGCAAG TGCATGCTGC TGGTCCTACC  1380
Cg123722  ---------- ---------- ---------- ---------- ---------- ----------  1124
Clustal Co                                                              1114
                1390      1400      1410      1420      1430      1440
        ....|....| ....|....| ....|....| ....|....| ....|....| ....|....|
Cg135193    TCACTGCCAT CCTCTGCAGC ATCATCAGGA TTCTCTGCAA CCGCAATGCC TGCTGCAGTC  1440
Cg123722  ---------- ---------- ---------- ---------- ---------- ----------  1124
Clustal Co                                                               1114
```

```
               1450      1460      1470      1480      1490      1500
           ....|....| ....|....| ....|....| ....|....| ....|....| ....|....|
Cg135193    CCAGCCTTCC CTTCTGCTCC TTCACACAAA AGACAACTGA TTCAATCAAA CGACAAGCCA  1500
Cg123722   ---------- ---------- ---------- ---------- ---------- ----------  1124
Clustal Co                                                                    1114
               1510      1520      1530      1540      1550      1560
           ....|....| ....|....| ....|....| ....|....| ....|....| ....|....|
Cg135193    TCTGCCCCCA TCAATATCCA GTTCAATTCT TCAGGCGAGG CATACAAGAG GAAGGGTCAG  1560
Cg123722   ---------- ---------- ---------- ---------- ---------- ----------  1124
Clustal Co                                                                    1114
               1570
           ....|....| ..
Cg135193    AACCGGGGGT GA 1572
Cg123722   ---------- -- 1124
Clustal Co              1114
```

miR172 binding site

```
Cg135193  1382 TCACTGCCAT CCTCTGCAGC ATCATCAGGA TTCTCTGCAA CCGCAATGCC TGCTGCAGTC  1440
```

B)

```
               10        20        30        40        50        60
           ....|....| ....|....| ....|....| ....|....| ....|....| ....|....|
Cg135193    MMLDLTLASS PDDTYKGSVE ASVNESGTSK SSVLNGDGSS NDDSCSNRVA AAGFRFGILK  60
Cg123722   MMLDLTLASS PDDTYKGSVE ASVNESGTSK SSVLNGDGSS NDESCSNRVA AAGFRFGILK  60
Clustal Co  ********** ********** ********** ********** **:******* **********  60

               70        80        90        100       110       120
           ....|....| ....|....| ....|....| ....|....| ....|....| ....|....|
Cg135193    DGDDEDDDLV EQEVPNDDDG VDDDGAFGRM GGLATKDLFP PPMMACVEGT LGANAAGIRA  120
Cg123722   DGDDEDDDLV EQEVPNDDDG VDDDGAFGRM GGLATKDLFP PPMMACVEGT LGANAAGIRA  120
Clustal Co  ********** ********** ********** ********** ********** **********  120

               130       140       150       160       170       180
           ....|....| ....|....| ....|....| ....|....| ....|....| ....|....|
Cg135193    VPFVPVGSSS SSAACRPQLV DLSFCRRDAA VRPEVRTLPQ QPPPPPPQQQ QPQQQVKKSR  180
Cg123722   VPFVPVGSSS SSAACRPQLV DLSFCRRDAA VRPEVRTLPQ QPPPPPPQQQ -PQHQVKRSR  179
Clustal Co  ********** ********** ********** ********** **********  **:***:**  179

               190       200       210       220       230       240
           ....|....| ....|....| ....|....| ....|....| ....|....| ....|....|
Cg135193    RGPRSRSSQY RGVTFYRRTG RWESHIWDCG KQIYLGGFDT AHAAARAYDR AAIKFRGIDA  240
Cg123722   RGPRSRSSQY RGVTFYRRTG RWESHIWDCG KQIYLGGFDT AHAAARAYDR AAIKFRGIDA  239
Clustal Co  ********** ********** ********** ********** ********** **********  239

               250       260       270       280       290       300
           ....|....| ....|....| ....|....| ....|....| ....|....| ....|....|
Cg135193    DINFNLSDYD DDMKLMSNLS KEEFVHILRR QSTGFSRGSS KYRGVTLHKC GRWEARMGQF  300
Cg123722   DINFNLSDYD DDMKLMSNLS KEEFVHILRR QSTGFSRGSS KYRGVTLHKC GRWEARMGQF  299
Clustal Co  ********** ********** ********** ********** ********** **********  299

               310       320       330       340       350       360
           ....|....| ....|....| ....|....| ....|....| ....|....| ....|....|
Cg135193    LGKKYIYLGL FDSEVEAARA YDKAAIRCNG REAVTNFDLS TYEGELISEA DTEGHNLDLS  360
Cg123722   LGKKYIYLGL CDSEVEAARA YDKAAIRCNG REAVTNFDLS TYEGELISEA DTEGHNLDLS  359
Clustal Co  **********  ********* ********** ********** ********** **********  358

               370       380       390       400       410       420
```

```
            ....|....| ....|....| ....|....| ....|....| ....|....| ....|....|
Cg135193      LSISQPASDS PKGDKTPNTV LIQSLSFDSP EVKKSRIDFP SSFSISQTCR PAEGRHGLPM  420
Cg123722    LSISQPASDS PKGD------ ---------- ---------- ---------- ----------  373
Clustal Co    ********** ****                                              372

                    430       440       450       460       470       480
            ....|....| ....|....| ....|....| ....|....| ....|....| ....|....|
Cg135193      WVTQANCFPS FEGSALDKRP ETKPIALPSL SWQVHAAGPT SLPSSAASSG FSATAMPAAV  480
Cg123722    ---------- ---------- ---------- ---------- ---------- ----------  373
Clustal Co                                                               372

                    490       500       510       520
            ....|....| ....|....| ....|....| ....|....| ....
Cg135193      PAFPSAPSHK RQLIQSNDKP SAPINIQFNS SGEAYKRKGQ NRG- 523
Cg123722    ---------- ---------- ---------- ---------- ---- 373
Clustal Co                                            372
```

C)

Cg135193 MMMLDLTLASSPDDTYKGSVEASVNESGTSKSSVLNGDGSSNDDSCSNRVAAAGFRFGILK 60
Cg123722 MMMLDLTLASSPDDTYKGSVEASVNESGTSKSSVLNGDGSSNDESCSNRVAAAGFRFGILK 60

Cg135193 DGDDEDDDLVEQEVPNDDDGVDDDGAFGRMGGLATKDLFPPPMMACVEGTLGANAAGIRA 120
Cg123722 DGDDEDDDLVEQEVPNDDDGVDDDGAFGRMGGLATKDLFPPPMMACVEGTLGANAAGIRA 120

NLS
Cg135193 VPFVPVGSSSSSAACRPQLVDLSFCRRDAAVRPEVRTLPQQPPPPPPQQQQPQQQ**VKKSR** 180
Cg123722 VPFVPVGSSSSSAACRPQLVDLSFCRRDAAVRPEVRTLPQQPPPPPPQQQ*PQHQ**VKRSR** 180

AP2-R1
Cg135193 RGPRSRSS**QYRGVTFYRRTGRWESHIWDCGKQIYLGGFDTAHAAARAYDRAAIKFRGIDA** 240
Cg123722 RGPRSRSS**QYRGVTFYRRTGRWESHIWDCGKQIYLGGFDTAHAAARAYDRAAIKFRGIDA** 240

AP2-R2
Cg135193 **DINFNLSDYD**DDMKLMSNLSKEEFVHILRRQSTGFSRGSS**KYRGVTLHKCGRWEARMGQF** 300
Cg123722 **DINFNLSDYD**DDMKLMSNLSKEEFVHILRRQSTGFSRGSS**KYRGVTLHKCGRWEARMGQF** 300

Cg135193 **LGKKYIYLGLFDSEVEAARAYDKAAIRCNGREAVTNFDLSTYEG**ELISEADTEGHNLDLS 360
Cg123722 **LGKKYIYLGLCDSEVEAARAYDKAAIRCNGREAVTNFDLSTYEG**ELISEADTEGHNLDLS 360

Cg135193 LSISQPASDSPKGDKTPNTVLIQSLSFDSPEVKKSRIDFPSSFSISQTCRPAEGRHGLPM 420
Cg123722 LSISQPASDSPKGD 374

miR172
Cg135193 WVTQANCFPSFEGSALDKRPETKPIALPSLSWQVHAAGPTSLPSSS**AASSGF**SATAMPAAV 480
Cg123722 374

Cg135193 PAFPSAPSHKRQLIQSNDKPSAPINIQFNSSGEAYKRKGQNRG- 523
Cg123722 374
