# Supplementary material for: SMRT and Illumina RNA-Seq Identifies Potential Candidate Genes Related to the Double Flower Phenotype and Unveils SsAP2 as a Key Regulator of the Double-Flower Trait in Sagittaria sagittifolia
Source: Int J Mol Sci. 2022 Feb 17;23(4):2240. doi: 10.3390/ijms23042240 (PMC8875719; doi:10.3390/ijms23042240)
Supplement: Supplementary file 1 [file ijms-23-02240-s001.zip › Supplementary Table 9-Screening and identification of transgenic Sagittaria sagittifolia.pdf]

1. Screening and identification of transgenic *S. sagittifolia*

pBI121 empty plasmids and restriction endonucleases from Figure S1 of the identified transgenic *S. sagittifolia* products amplified by PCR using positive expression vectors are shown. The restriction endonuclease digestion verified that the correct products were produced via PCR amplification.

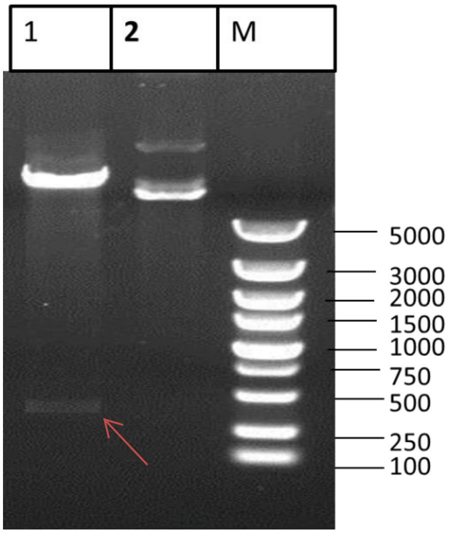


**Figure S1.** Electrophoretic pattern of PCR products of the expression vectors digested by *Xba*I and *Xma*I. Lane M: DNA marker; Lane 1: plasmid digested by *Xba*I and *Xma*I; Lane 2: plasmid DNA.

2. Screening of transgenic *S. sagittifolia* plants

All the growth conditions for the plants remained the same except for the use of Kan to select the transformants. The screening of transgenic plants on media that contained 40 mg/L, 50 mg/L, 60 mg/L, 70 mg/L, and 80 mg/L Kan is shown in Table S1:

**Supplementary Table 9-1.** The influence of different antibiotic concentrations on the conversion efficiency.

| Kan concentration (mg/L) | 40 | 50 | 60 | 70 | 80 |
|---|---|---|---|---|---|
| Green seedling rate (%) | 65 | 20.4 | 8.9 | 2.2 | 0.5 |

The effects of different concentrations of antibiotic on the transformation efficiency indicate that the optimal concentration for screening was 50 mg/L. Therefore, when the transgenic plants were cultured in MS media that contained 50 mg/L of kanamycin, more of them survived. The non-transgenic seedlings gradually yellowed and died, while the green transgenic plants grew normally.

3. The rapid identification of transgenic *S. sagittifolia*

The transgenic seedlings were identified by PCR, with non-transgenic *S. sagittifolia* as the control. A DNA template and universal primers were used when constructing the transgenic expression vector. After digestion with *Bam*H1, a bright band in Lane 1 of approximately 350 bp was obtained from the transgenic-positive plants and was consistent with the expected result (Figure S2). The target bands of PCR amplification are positive bands, and the plants detected are transgenic plants.

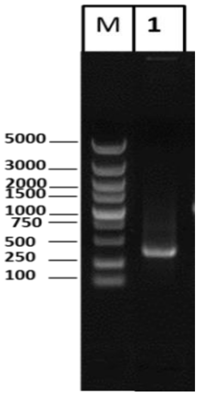


**Figure S2.** Identification of the PCR products from transgenic plants by *Bam*H1 restriction endonuclease digestion.
