# Supplementary figures and images for: SMRT and Illumina RNA-Seq Identifies Potential Candidate Genes Related to the Double Flower Phenotype and Unveils SsAP2 as a Key Regulator of the Double-Flower Trait in Sagittaria sagittifolia
Source: Int J Mol Sci. 2022 Feb 17;23(4):2240. doi: 10.3390/ijms23042240 (PMC8875719; doi:10.3390/ijms23042240)

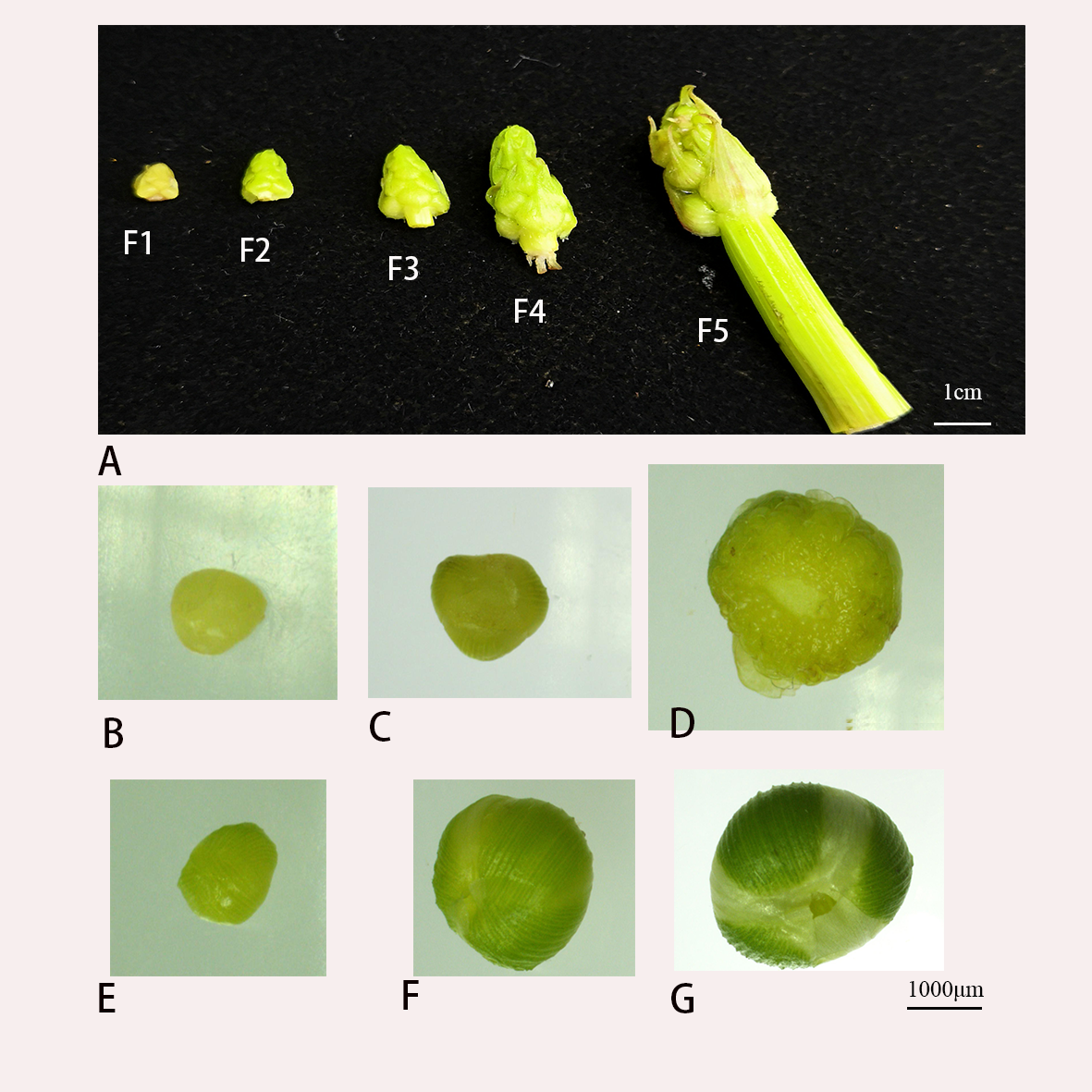

Supplement: Supplementary file 1 [file ijms-23-02240-s001.zip › suppletary Figure1. Observations of the external characteristics of female and male flower (bud) differentiation..tif]

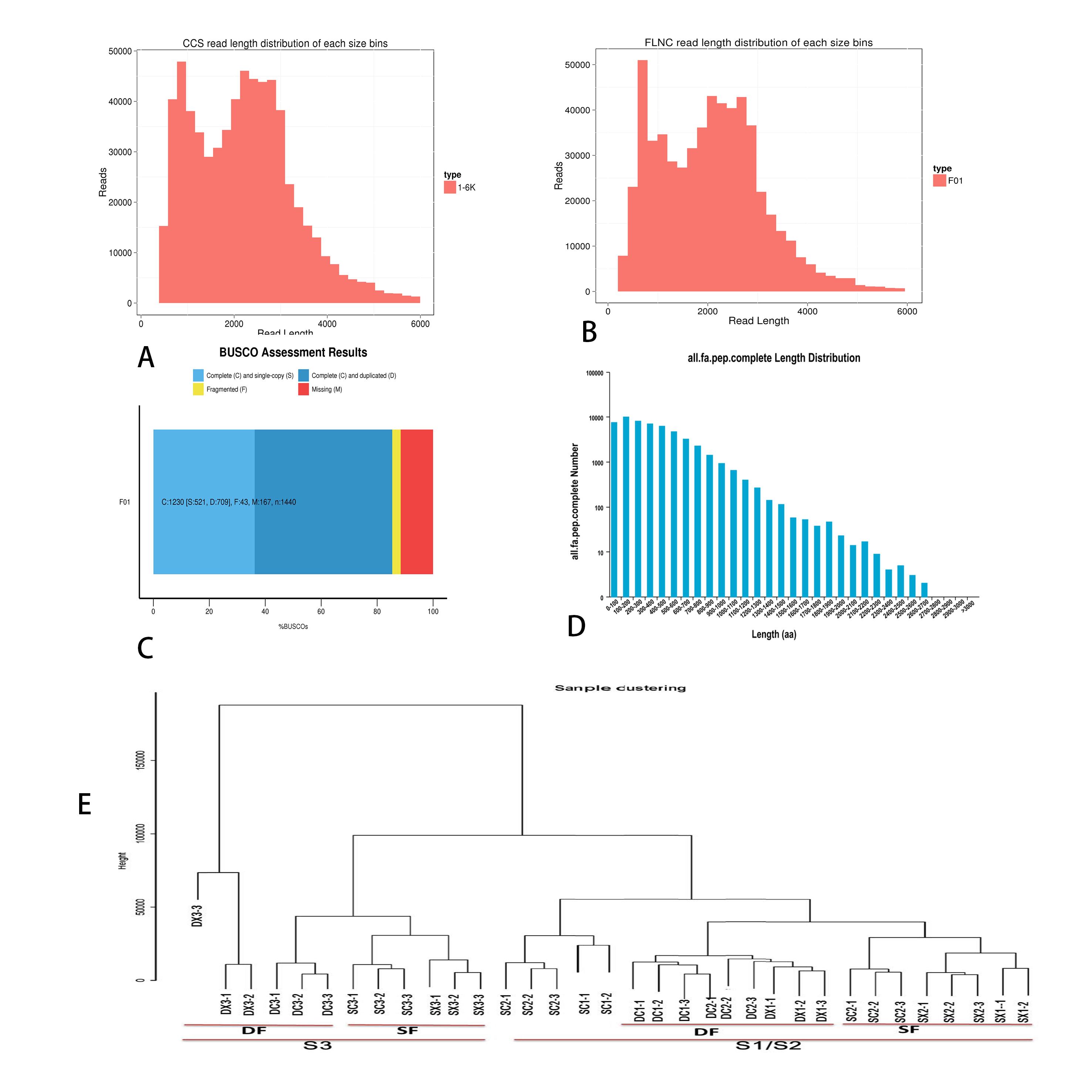

Supplement: Supplementary file 1 [file ijms-23-02240-s001.zip › suppletary Figure2-. Quality control of the sequencing data and Sample system cluster diagram..tif]
